# Supplementary material for: Traject3d allows label-free identification of distinct co-occurring phenotypes within 3D culture by live imaging
Source: Nat Commun. 2022 Sep 9;13:5317. doi: 10.1038/s41467-022-32958-x (PMC9463449; doi:10.1038/s41467-022-32958-x)
Supplement: Supplementary file 1 — Supplementary Information File [file 41467_2022_32958_MOESM1_ESM.pdf]

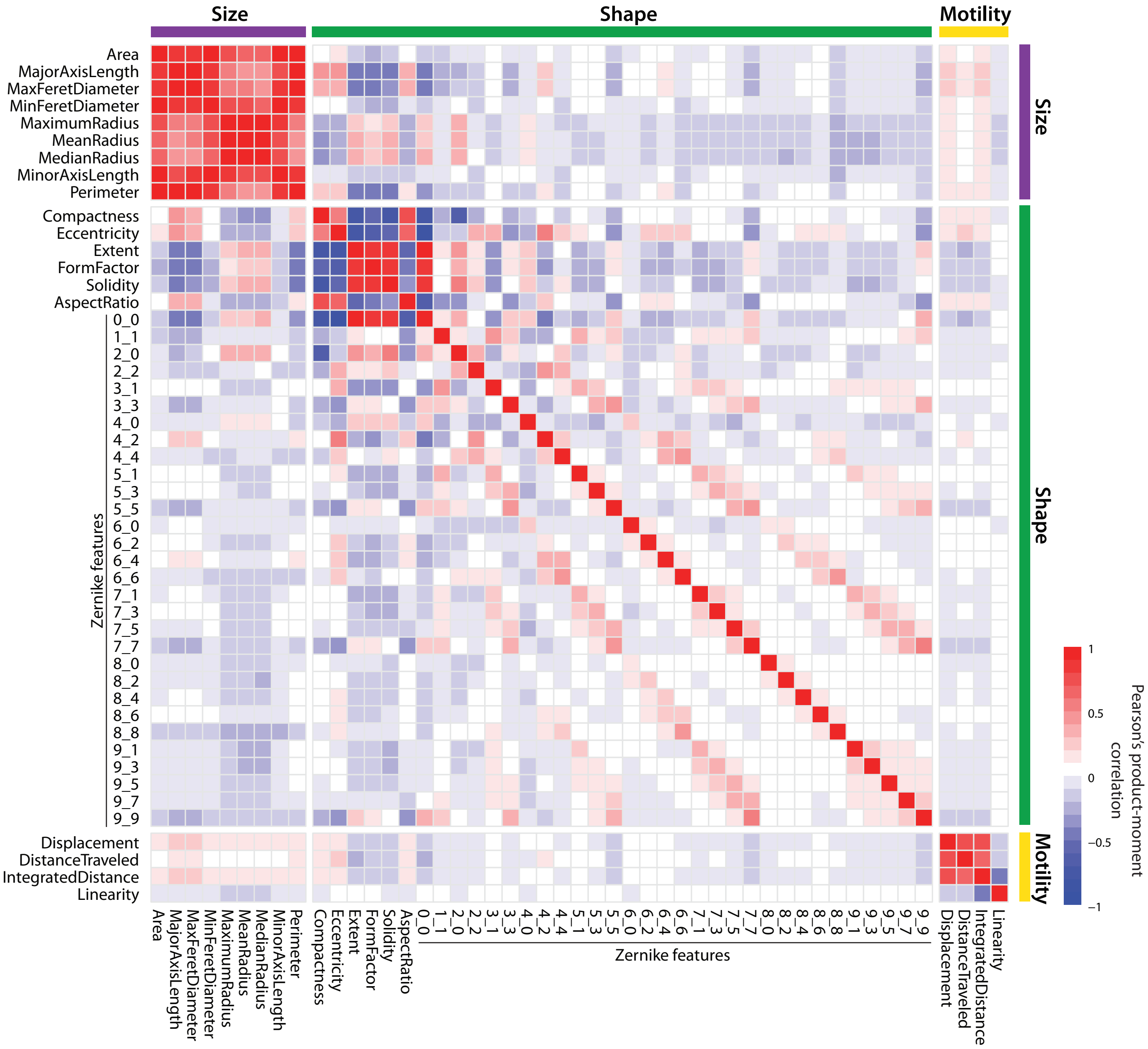

Supplementary Figure 1

Supplementary Figure 1. Correlation matrix of parameters measured by Cell Profiler.

Parameter correlation matrix. Correlation matrix of size (purple), shape (green), and motility (yellow) parameters as measured by CellProfiler. Pearson's product moment correlation shown in blue-red scale. Analysis performed on a total of 1,662,584 spheroids from 22 cell lines (see Supplementary Table 2). n=between 1 and 3 independent experiments, each with between 3 and 6 wells/condition, between 21,023 and 132,876 spheroids/condition quantified in total.

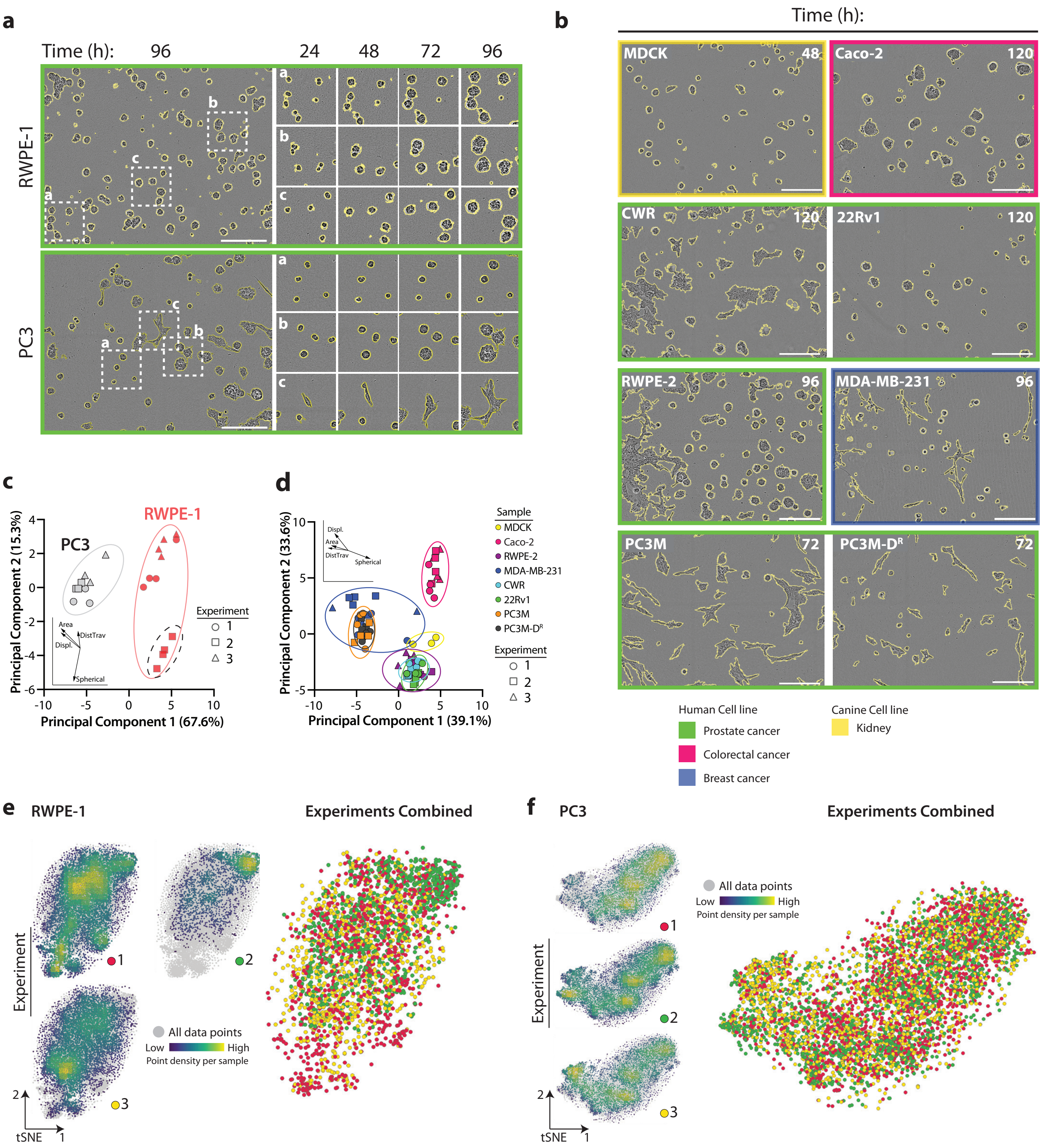

Supplementary Figure 2

Supplementary Figure 2. Heterogeneous phenotypes of spheroids occur in parallel within multiple cell lines.

a. Representative phase images of RWPE-1 (non-tumourigenic) and PC3 (metastatic) prostate cell line spheroids at 96 hours. Higher magnification of boxed regions (a-c) is shown. n=3 independent experiments. Scale bar, 100μm.

b. Phase images of spheroids formed from human prostate (CWR, 22RV1, RWPE-2, PC3M and PC3M-D^R), colorectal (Caco-2) and breast (MDA-MB-231) cancer cell lines. Image of Madin-Darby Canine Kidney spheroids is shown (MDCK). Frames coloured to indicate tissue of origin. MDCK: n=1 independent experiment; Caco-2, RWPE-2, MDA-MB-231: representative of n=3 independent experiments; and CWR, 22Rv1, PC3M, PC3M-DR: representative of n=2 independent experiments. Scale bar, 100μm.

c. PCA of PC3 and RWPE-1 spheroids replicates exemplified in **(a)**. Analysed data was comprised of every spheroid identified in each timepoint (image frame) of the experiment. Measurements were averaged by well (each point representing one well), where experiment is indicated by point shape, and cell line by colour. Dashed ellipse indicates a potential batch effect. PC3: n=3 independent experiments, each with 3 wells. RWPE-1: n=3 independent experiments, each with 4 wells. Total quantified spheroids stated in Supplementary Table 2.

d. PCA of spheroids replicates from cell lines exemplified in **(b)**. Analysed data was comprised of every spheroid identified in each timepoint (image frame) of the experiment. Measurements were averaged by well (each point a well), where experiment is indicated by shape, and cell line by colour. Ellipse per cell line around respective points. MDCK: n=1 independent experiment, 3 wells. Caco-2, RWPE-2, MDA-MB-231: n=3 independent experiments, each with 4 wells/condition. CWR, 22Rv1, PC3M, PC3M-DR: n=2 independent experiments, each with 4 wells/condition. Total quantified spheroids/condition stated in Supplementary Table 2.

e-f. t-SNE of RWPE-1 and PC3 spheroids exemplified in **(a)**. Analysed data was comprised of every spheroid identified in each image frame of the experiment. Plot

points coloured by experiment, and by the per experiment point density (purple to yellow). Total spheroids quantified in Supplementary Table 2. t-SNE analysis performed independently on 20,000 objects per cell line subsampled via GeoSketch, with iterations; 5,000, theta; 0.1, perplexity; 230.

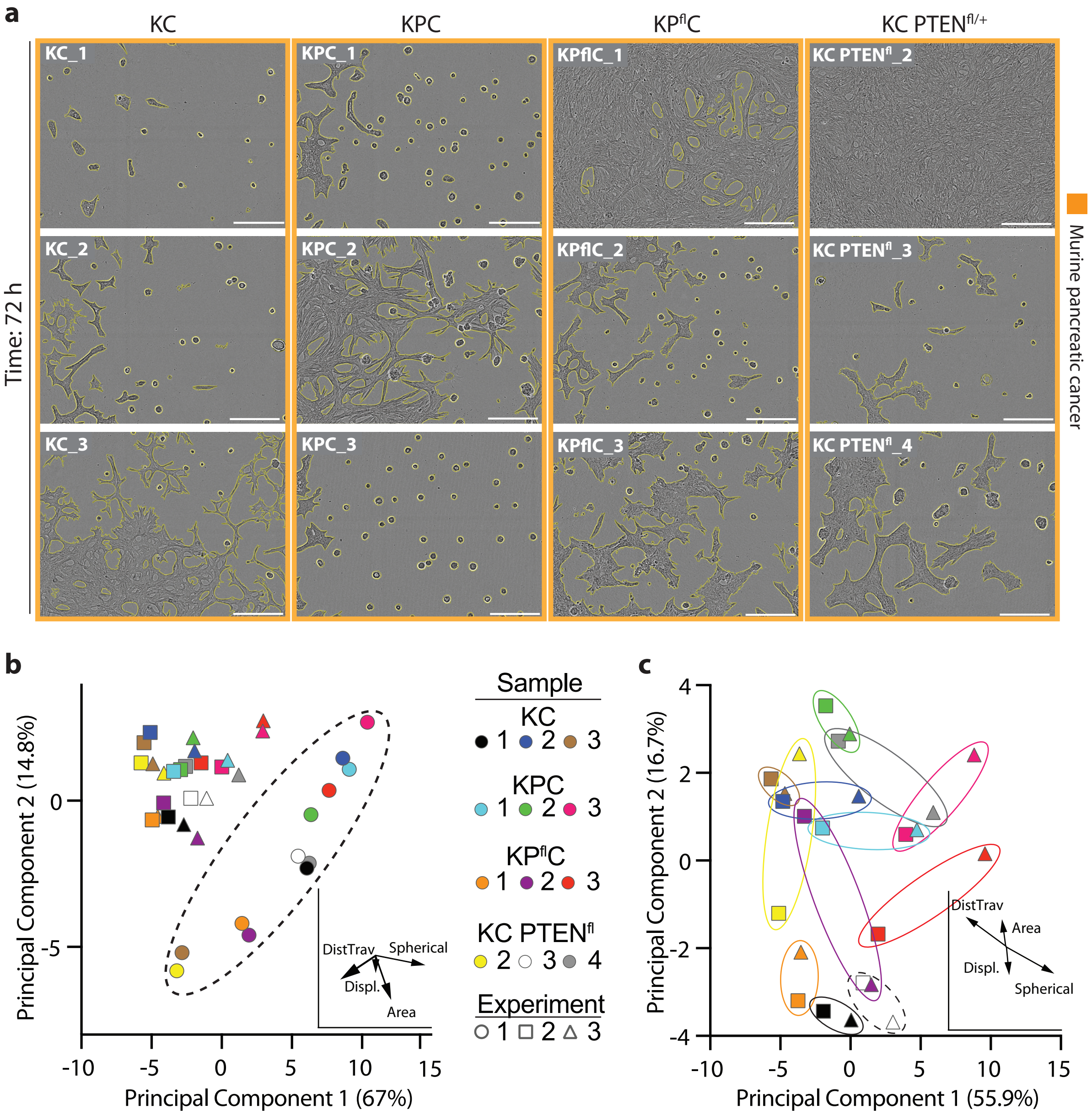

Supplementary Figure 3

Supplementary Figure 3. Heterogeneous phenotypes of spheroids occur in parallel within mouse tumour-derived organoids.

a. Phase images of spheroids from different genetically engineered murine models of pancreatic ductal adenocarcinoma (PDAC): *Pdx1*-Cre, LSL-*Kras*$^{G12D/+}$ (KC), *Pdx1*-Cre, LSL-*Kras*$^{G12D/+}$, LSL-*Trp53*$^{R172H/+}$ (KPC), *Pdx1*-Cre, LSL-*Kras*$^{G12D/+}$, LSL-*Trp53*$^{fl/+}$ (KPflC) and KC *Pten*$^{fl/+}$ (KC PTEN^{fl}). Note lack of homogeneity within and between cell lines. Cell lines derived from 3 different mice are shown for each model. Representative of n=3 independent experiments. Scale bar, 100μm.

b-c. PCA of spheroid experiments from cell lines shown in **(a)**. Measurements were averaged by experiment (each point an experiment), which is indicated by point shape, and cell line is represented by point colour. Ellipse per cell line around respective points. Clustering of Experiment 1 in **(b)** indicates a batch effect, highlighted by a dashed ellipse. This was resolved upon exclusion and reanalysis, **(c)**, indicated by improved clustering of points by Sample. Total spheroids/condition shown in Supplementary Table 2. **(b)**: n=3 independent experiments, each with between 3 and 6 wells/condition, between 39,715 and 85,515 spheroids/condition quantified in total. **(c)**: Reanalysed subset of data from **(b)**. n=2 independent experiments, each with between 3 and 6 wells/condition, between 30,495 and 62,344 spheroids/condition quantified in total.

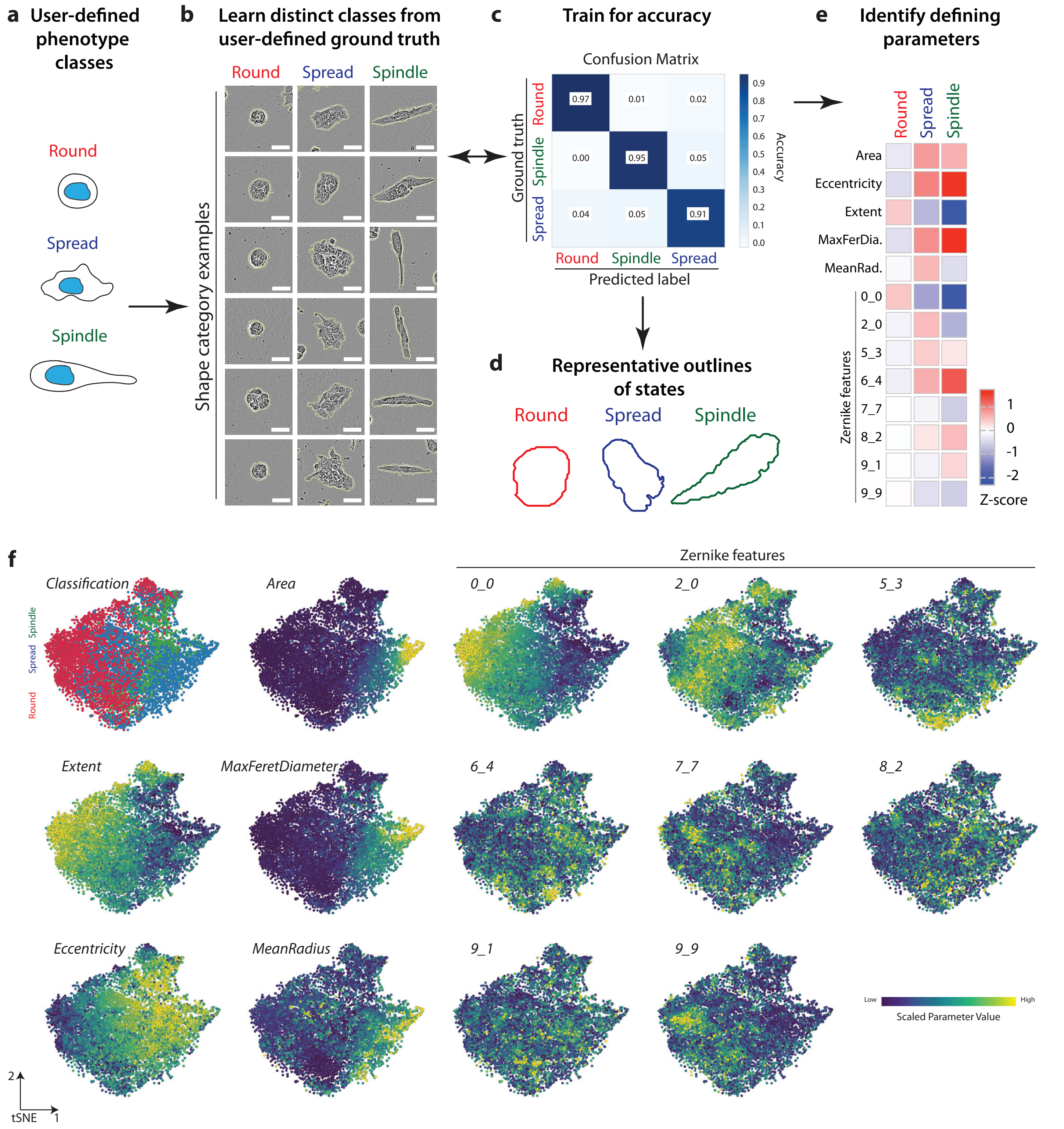

Supplementary Figure 4

Supplementary Figure 4. Generation and characterisation of user-defined classification of heterogeneous phenotypes.

a. Schema, classification of PC3 spheroids into 3 user-defined states: Round, Spread and Spindle.

b. Example phase images of PC3 spheroids, overlaid with outlines (yellow), used to classify spheroids into Round, Spread and Spindle states. Scale bar, 50μm.

c. Confusion matrix generated by CellProfiler Analyst to indicate fidelity of predicted class to the true user classification. A Fast Gentle Boosting machine learning model was used to define Round (97%), Spread (91%) and Spindle (95%) states of PC3 spheroids. Note high fidelity (%) of the machine learning classification, as defined by concordance between manual user classification during model training and subsequent classification using the model.

d. Schema, computationally selected representative outlines of Round, Spread and Spindle states.

e. Heatmap shows mean of size, shape and movement measurements generated by CellProfiler for Round, Spread and Spindle states. n=3 independent experiments, each with 3 wells/condition. 213,920 spheroids imaged in total.

f. t-SNE visualisation of parental PC3 and sublines, corresponding to analysis from Figure 2b-c. Plot points coloured by user-defined (Round, Spread, Spindle) state classifications, and by Z-score normalised value for shown measurements of spheroid morphology (purple to yellow). Total spheroids/condition listed in Supplementary Table 3. t-SNE analysis performed on 20,000 objects subsampled via GeoSketch algorithm, with iterations; 2,000, theta; 0.5, perplexity; 50.

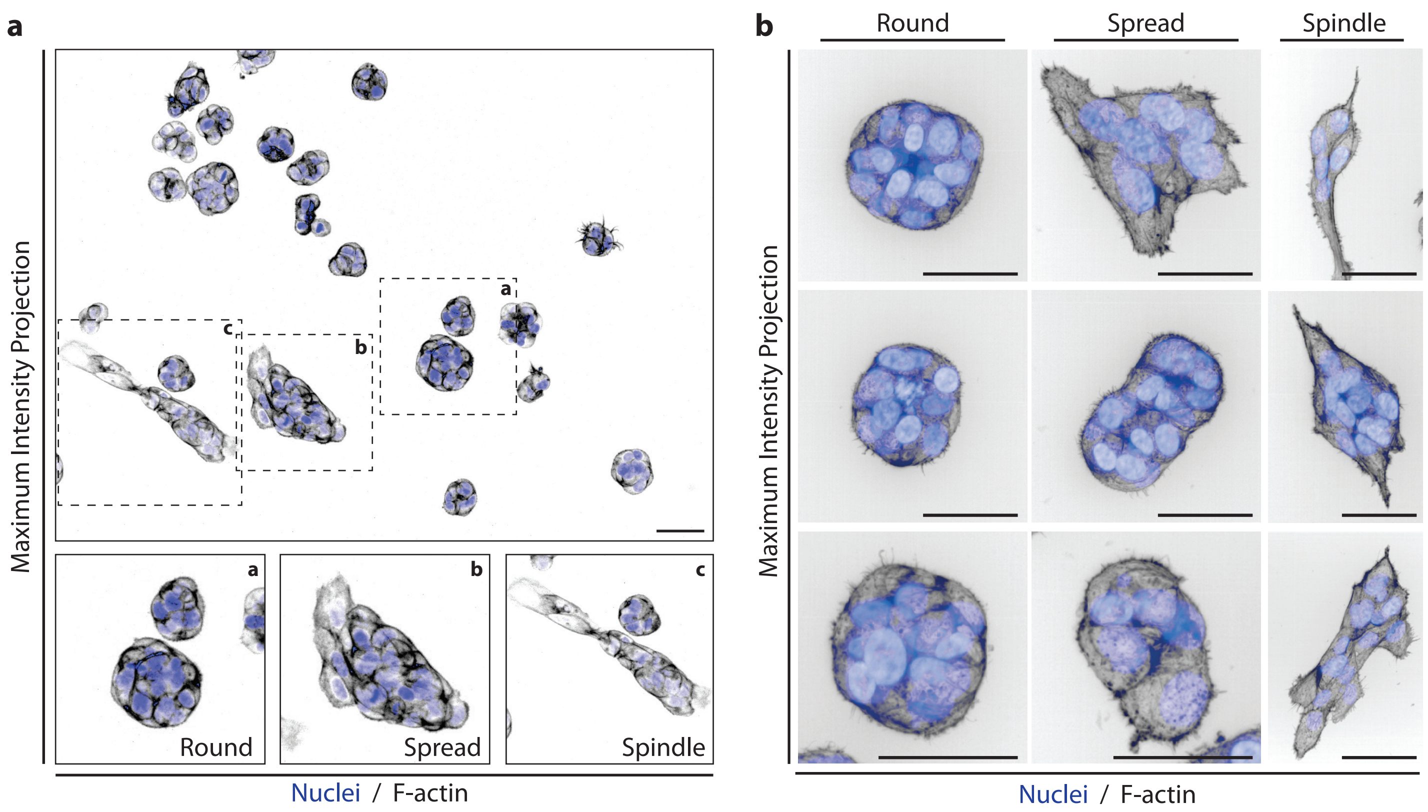

Supplementary Figure 5

Supplementary Figure 5. Maximum intensity images of PC3 spheroids

a-b. Examples of PC3 spheroids that were fixed and stained with Hoechst (nucleii) and Alexa Fluoro 568 Phalloidin (F-actin). Samples were imaged on an Opera Phenix™ High Content analysis system and 43 z-slices were used to generate maximum intensity images. Zoomed images of boxed regions in (a) show Round, Spread and Spindle phenotypes. Images are representative of n=3 independent experiments, each with 3 wells/condition. Scale bars, 50μm.

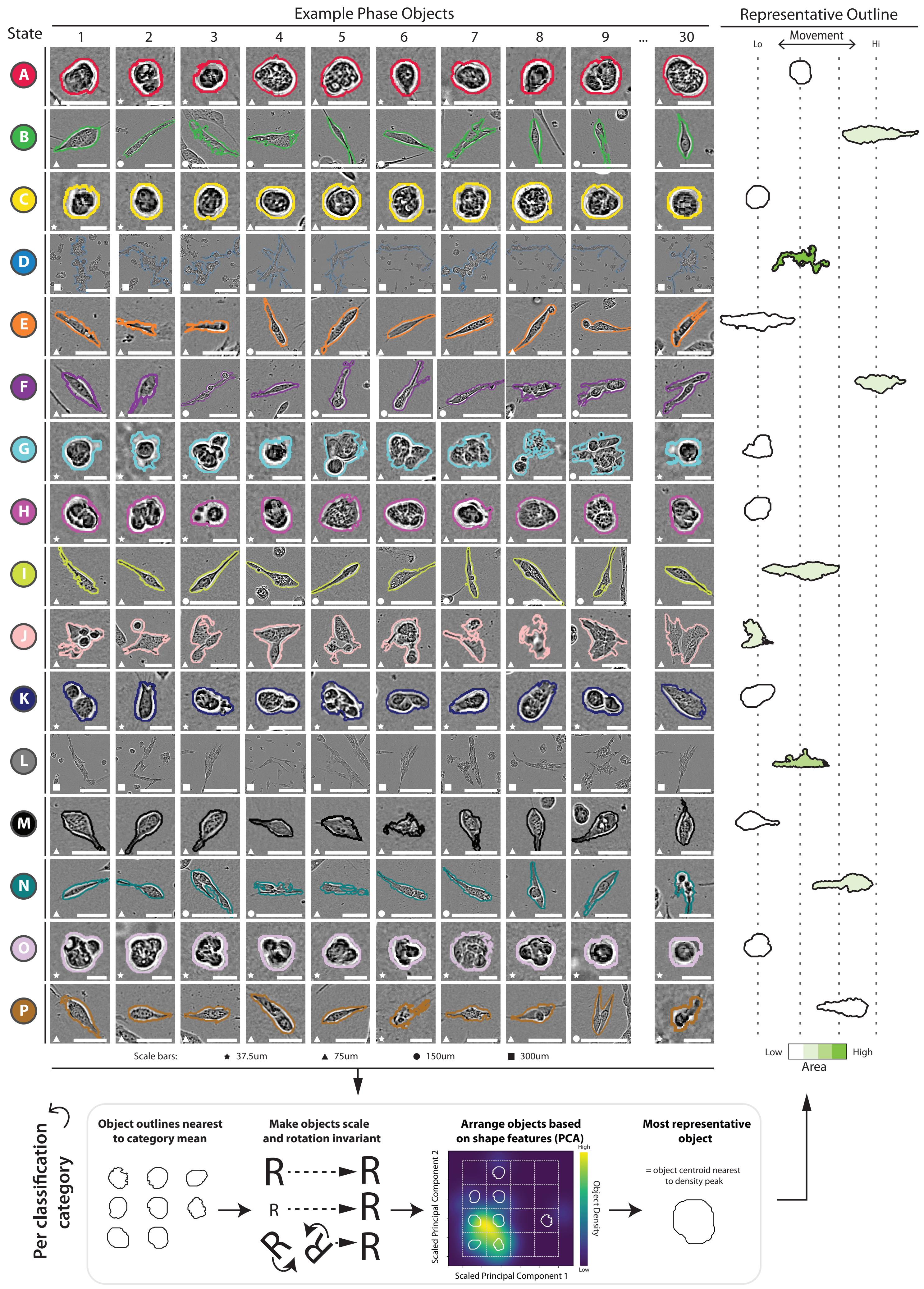

Supplementary Figure 6

Supplementary Figure 6. Selection of spheroids outlines to represent states.

Schema, selection of spheroid outlines to represent data-driven states. Thirty spheroids were selected for each state, based on Euclidian distance from mean morphological measurements of the group – ten phase images shown per group. Scale bars: 37.5μm (star), 75μm (triangle), 150μm (circle), and 300μm (square). The outlines of these spheroids were analysed to make them scale and rotation invariant, before arranging in 2-dimensional space using PCA. The spheroid outline nearest to the density peak across the two dimensions was selected. The outline selected for each state is shown, arranged by movement, and coloured by average area (green colour scale).

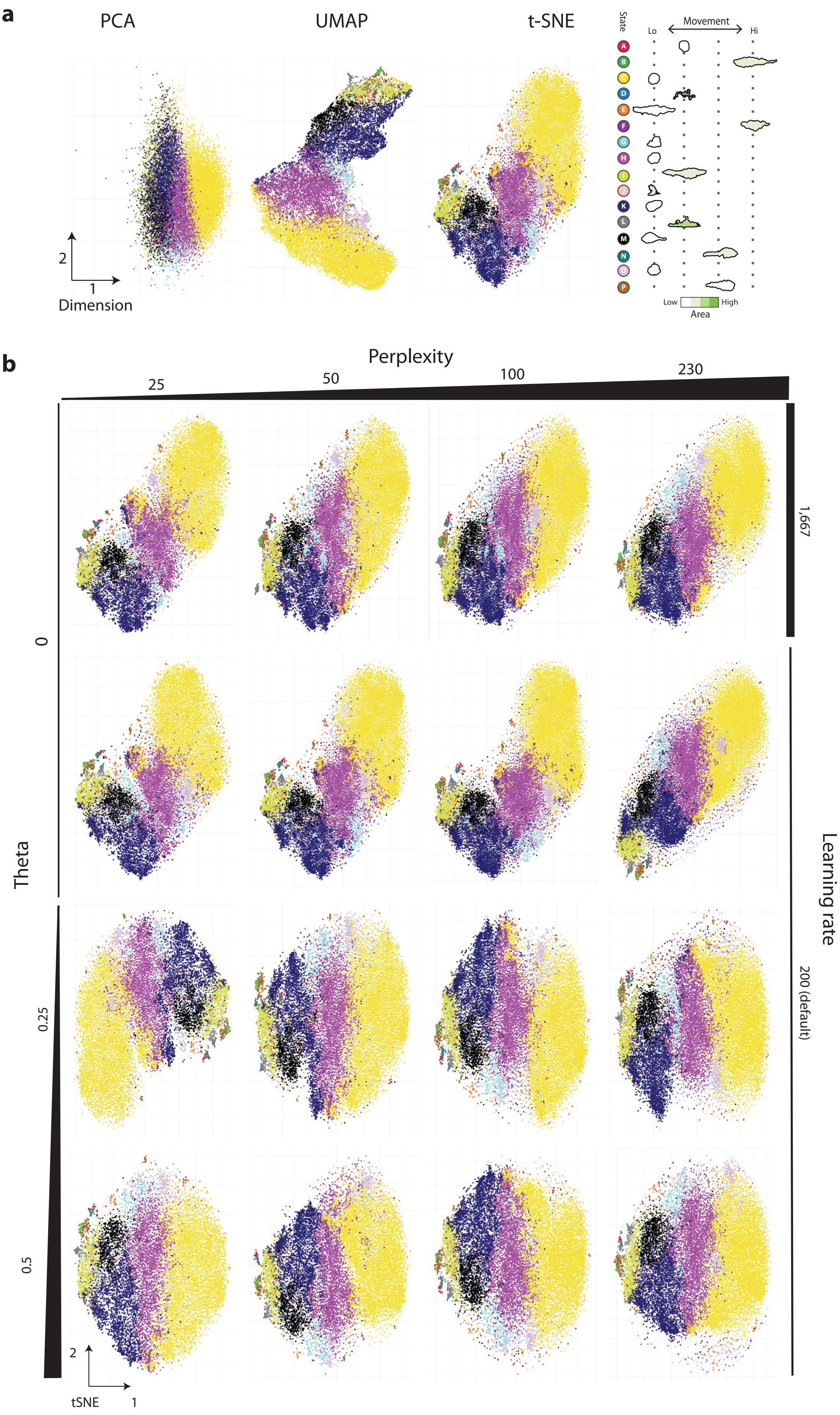

Supplementary Figure 7

Supplementary Figure 7. Dimensionality reduction optimisation.

a. Dimensionality reduction via PCA, UMAP, and t-SNE was performed on 20,000 randomly sampled spheroids from parental PC3 and variant sublines. Points coloured by data-driven state shown on right: representative spheroid outlines, arranged by average movement, and coloured by area (green colour scale). PCA and UMAP performed using default parameters. t-SNE performed with iterations; 5000, theta; 0, perplexity; 50, Learning Rate; 200. Total spheroids/condition listed in Supplementary Table 3.

b. Comparison of t-SNE visualisations resulting from varying perplexity, theta, and learning rate parameters. Parameters as stated in figure. Learning rate = 1,667 used based on guidelines in the literature[50]. Analysis performed on 20,000 randomly sampled spheroids from parental PC3 and variant sublines, see Supplementary Table 3 for total spheroids/condition. Points coloured by state, as in **(a)**. Note that varying parameters as above has minimal effect on overall distribution.

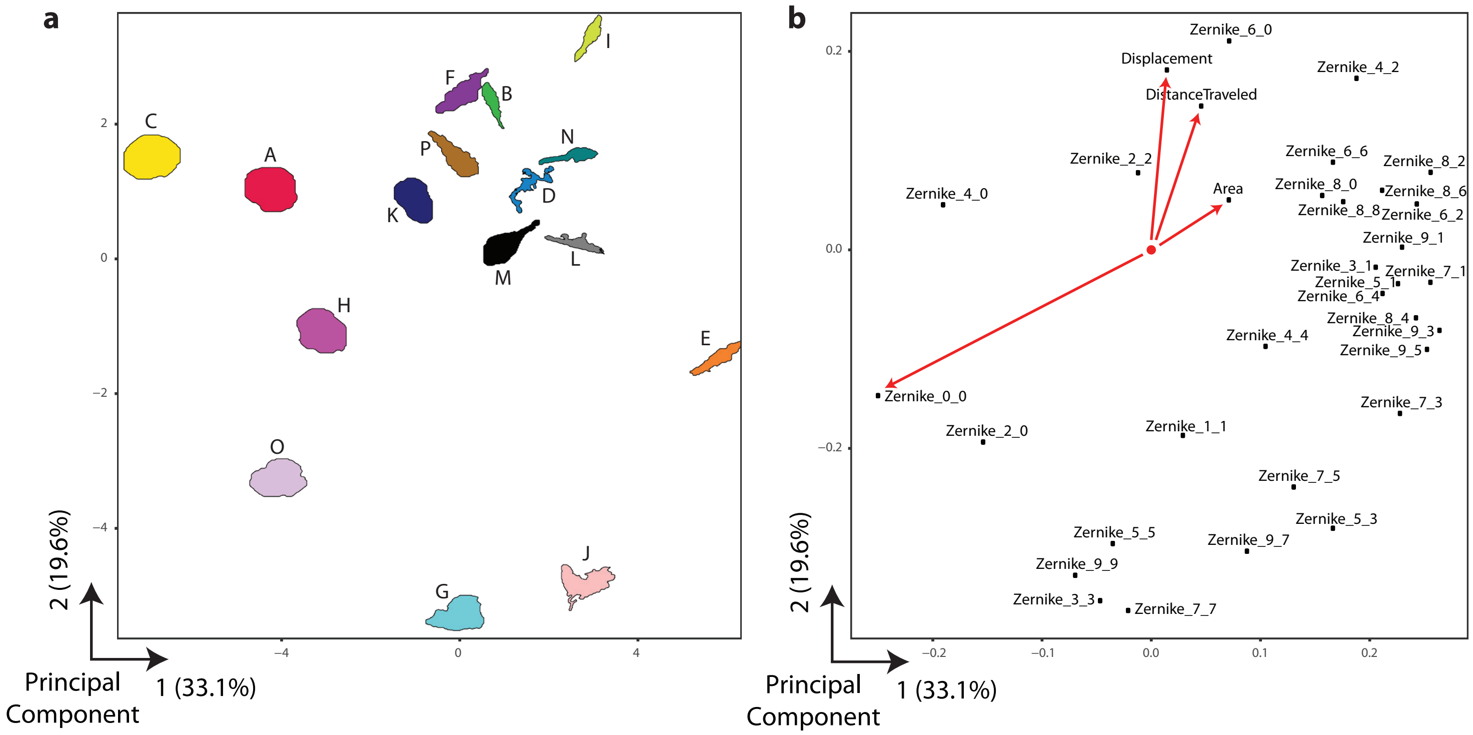

Supplementary Figure 8

Supplementary Figure 8. Definition of the phenotype state space.

a-b. PCA was performed on the mean measurements of size, shape, and movement, in order to arrange data-driven states in 2-dimensional space based on. **(a)** Example outline representing each state was projected onto the first two Principal Components, with loadings for the same analysis shown in **(b)**. Analysis performed on all 2,532,154 spheroids from cell lines in Supplementary Table 3.

a

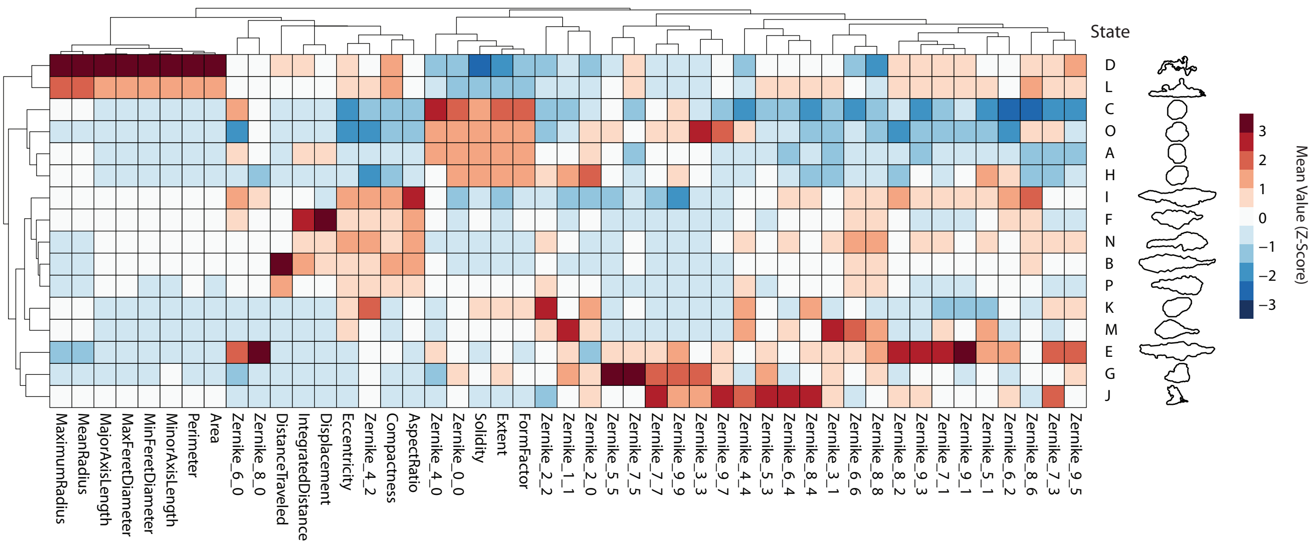

Supplementary Figure 9

Supplementary Figure 9. Measurements underpinning data-driven state classifications.

a. Heatmap showing mean normalised (Z-score) value of shape, size, and motility features for each data-driven state classification, coloured blue to red. A representative spheroid outline is shown for each classification.

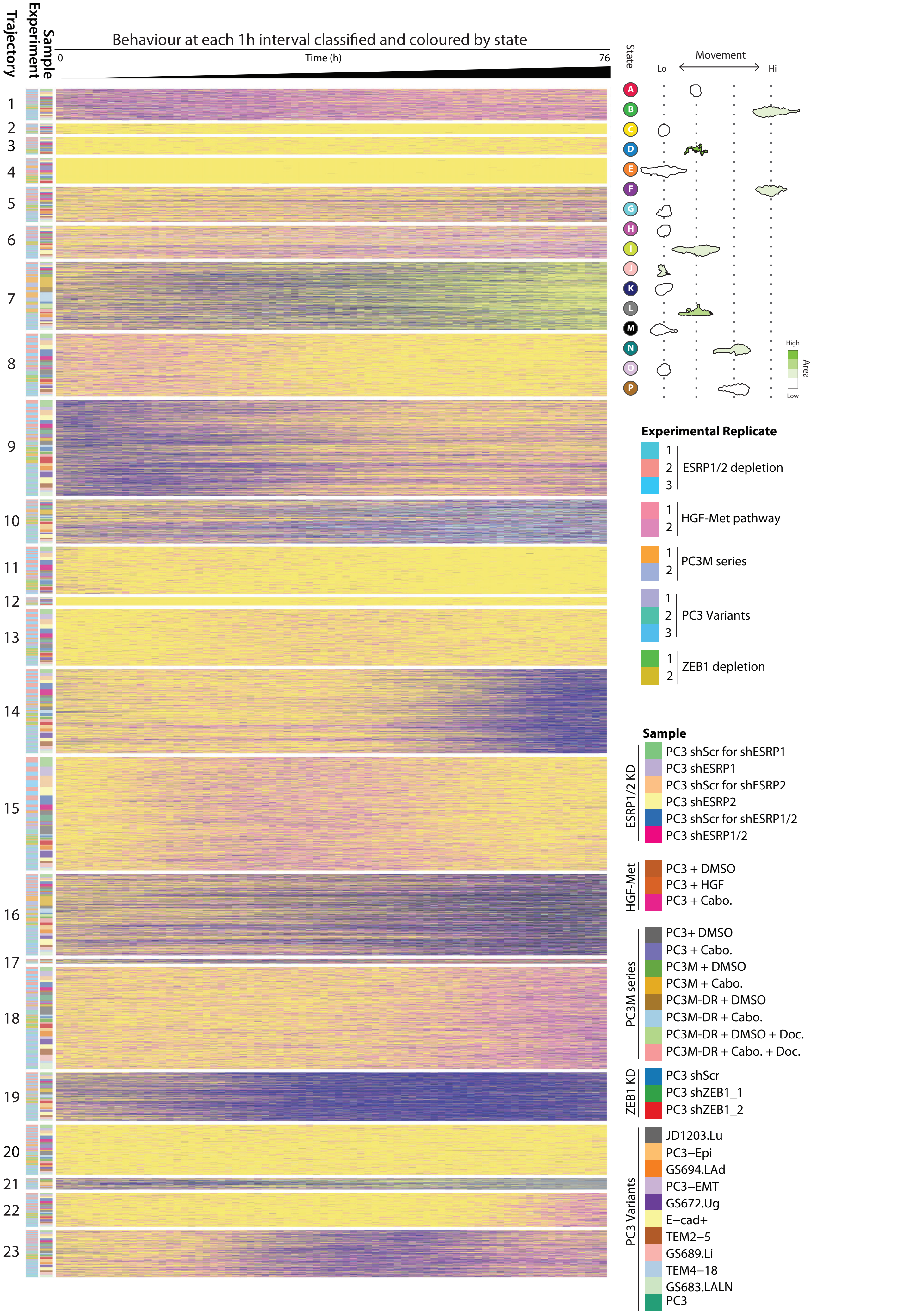

Supplementary Figure 10

Supplementary Figure 10. Heatmap of identified patterns of data-driven state change over time.

Heatmap of state classification over time for spheroids continuously tracked across all datasets, in which each row is a tracked spheroid and each column a timepoint (images taken hourly). The data at each point in the heatmap is coloured by data-driven state classification. Representative outline shown for each state, arranged by average movement, and coloured by average size (green colour scale). Rows are ordered by trajectory classification (recurring pattern of state change over time) and are annotated with cell line and experimental replicate. n=either 2 or 3 independent experiments, and each with either 3 or 4 wells/condition. Total spheroids/condition after the filtering steps required in order to retain only well-tracked spheroids listed in Supplementary Table 5.

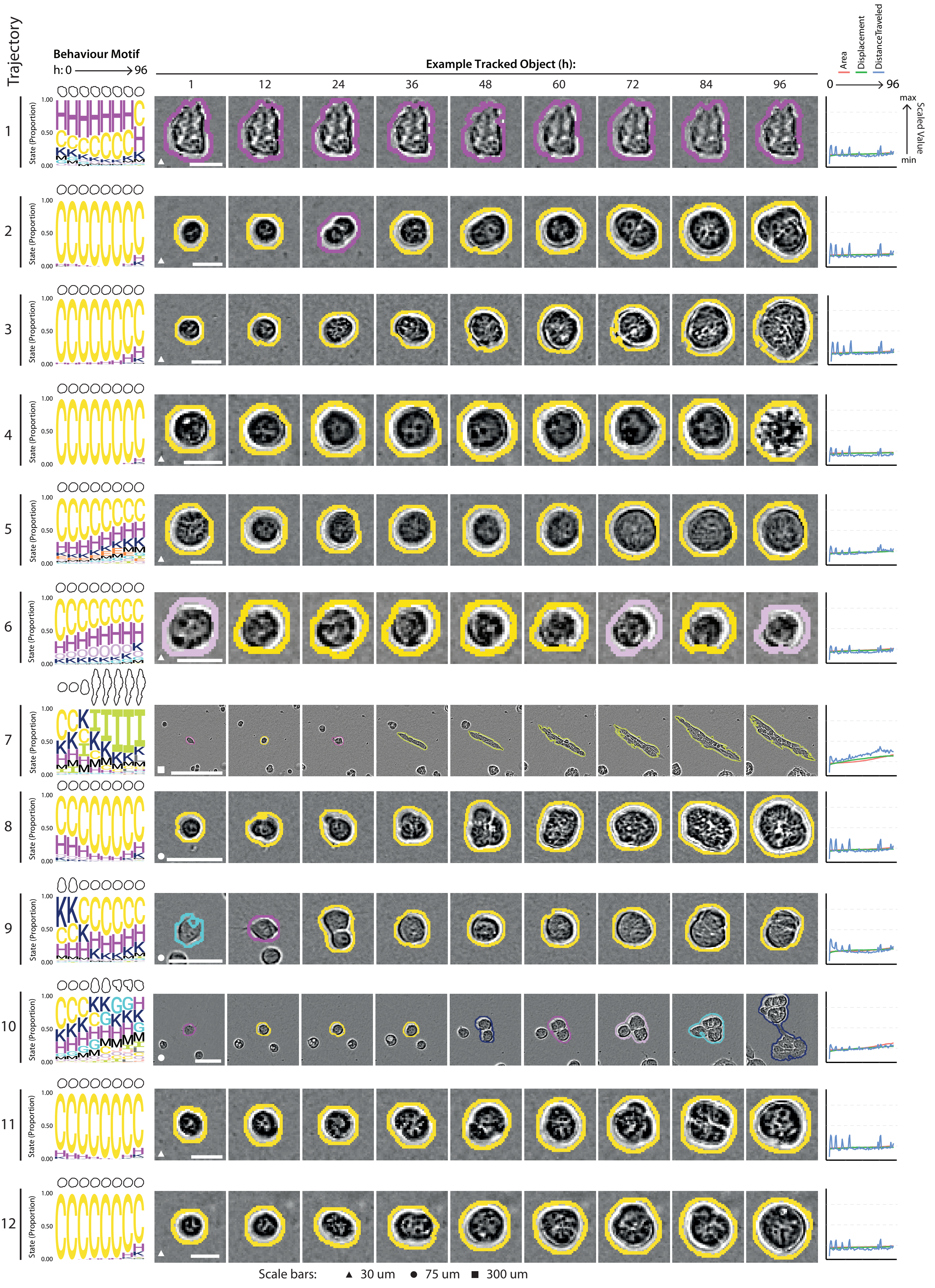

Supplementary Figure 11A

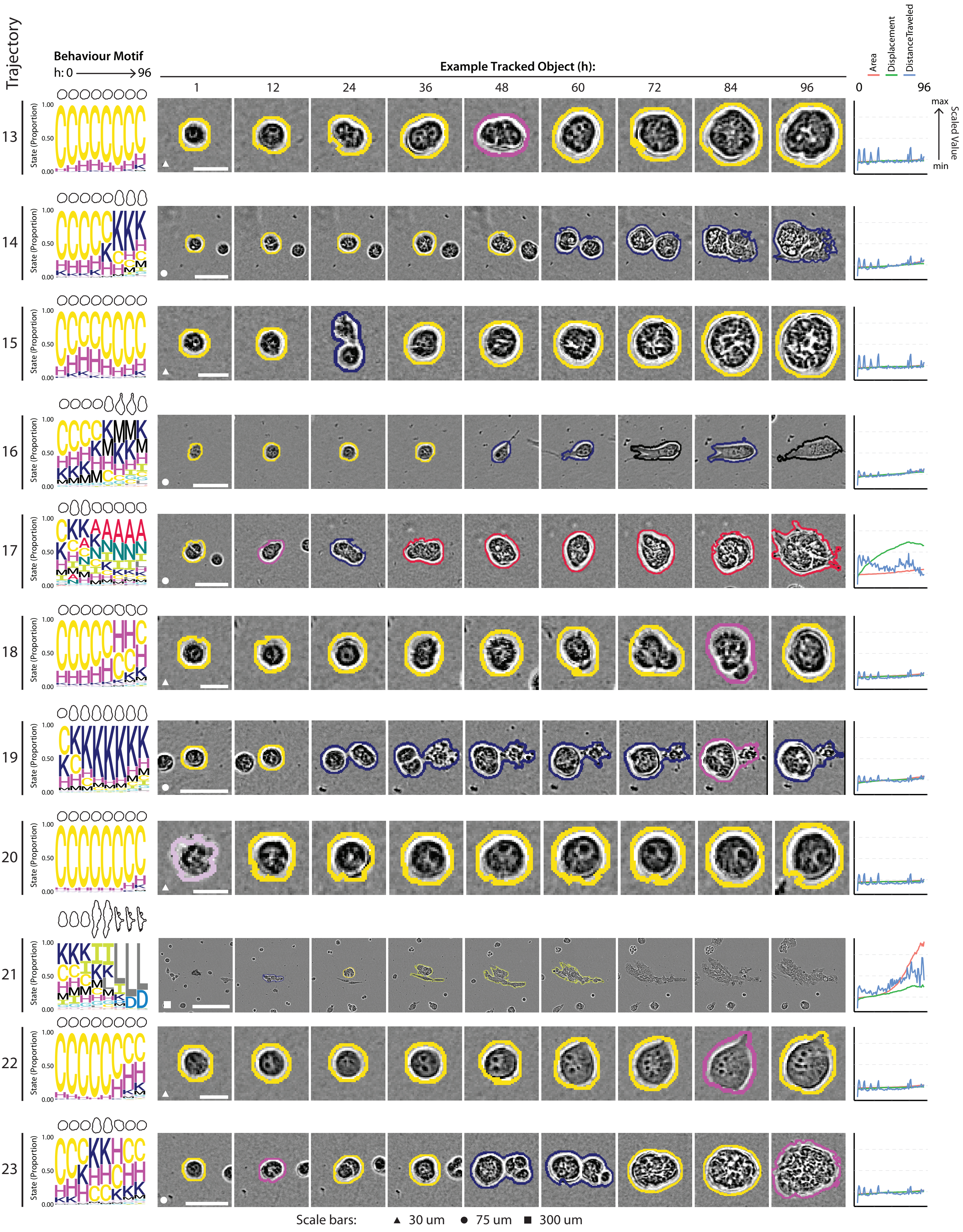

Supplementary Figure 11B

Supplementary Figure 11. Visualisation of the average behaviours within distinct trajectories.

A and B. Trajectory visualisation. Colours in the figure correspond to the previously identified states. Behaviour motif depicting frequency (proportion) of states in 12-hour time intervals, with outline of the most abundant state shown at top. Using the most frequent state at each timepoint, a spheroid was selected to represent the trajectory. Phase contrast images of these are shown, with outline colour indicating state classification at given timepoint. Scale bars, 30μm (triangle), 75μm (circle), and 300μm (square). Line plot showing mean values over time (Z-score) for spheroid Area (red), Displacement (green), and Distance Travelled (blue). Trajectories appearing in main figures – 6, 7 and 21 (Figure **3c-e**), 3 and 19 (Figure **5j and k**) and 16 and 17 (Figure **6f** and **g**) – are shown again for completeness.

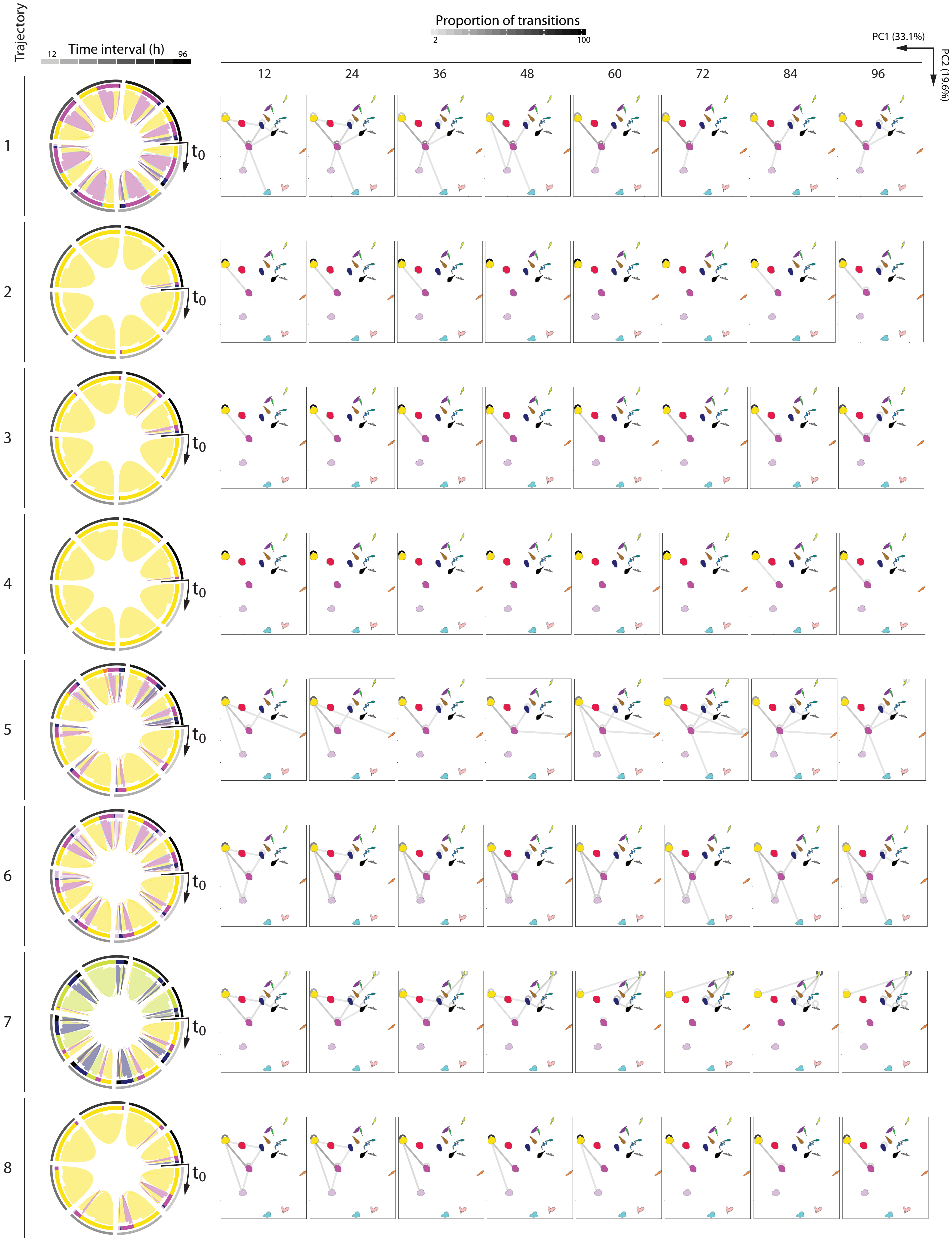

Supplementary Figure 12A

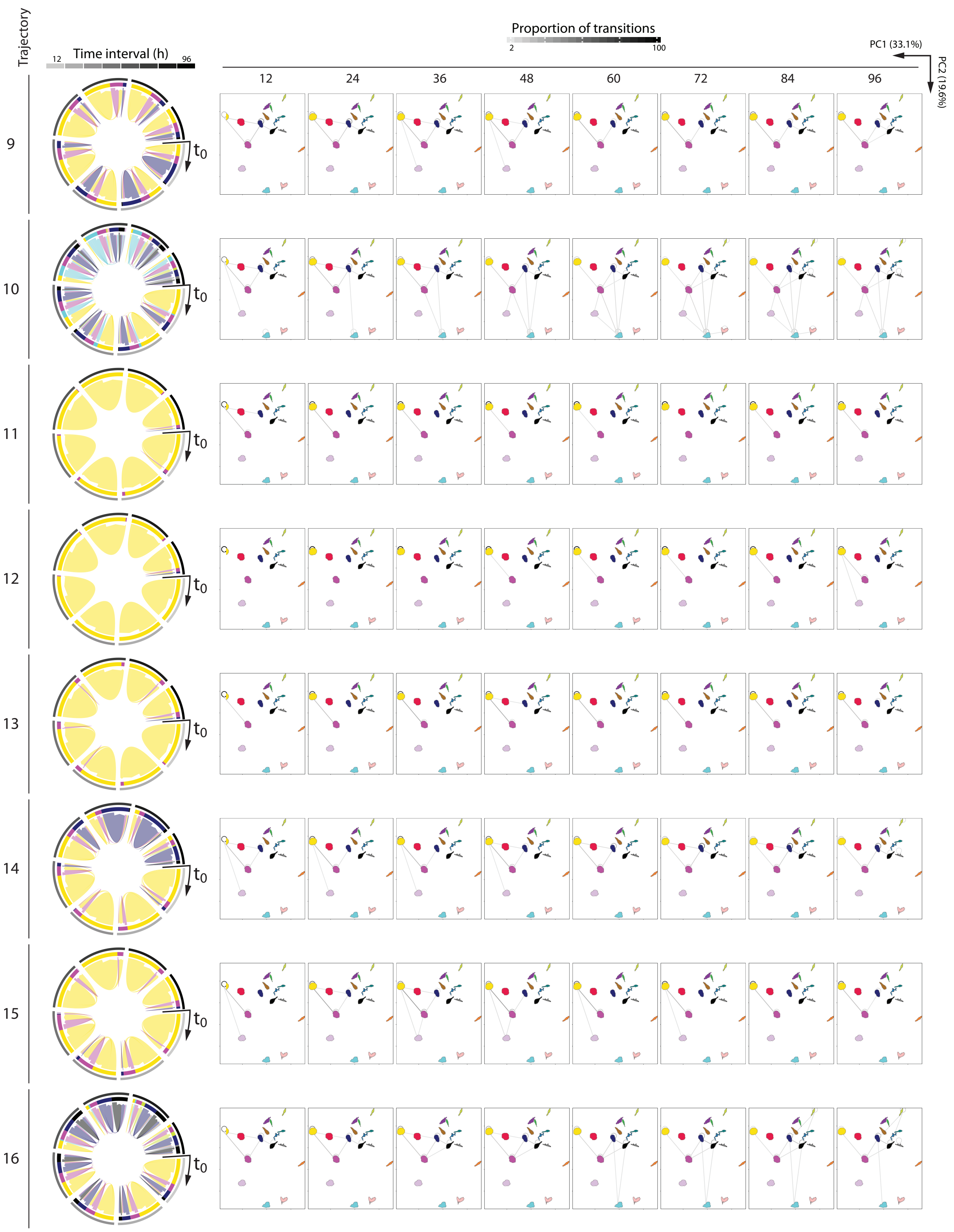

Supplementary Figure 12B

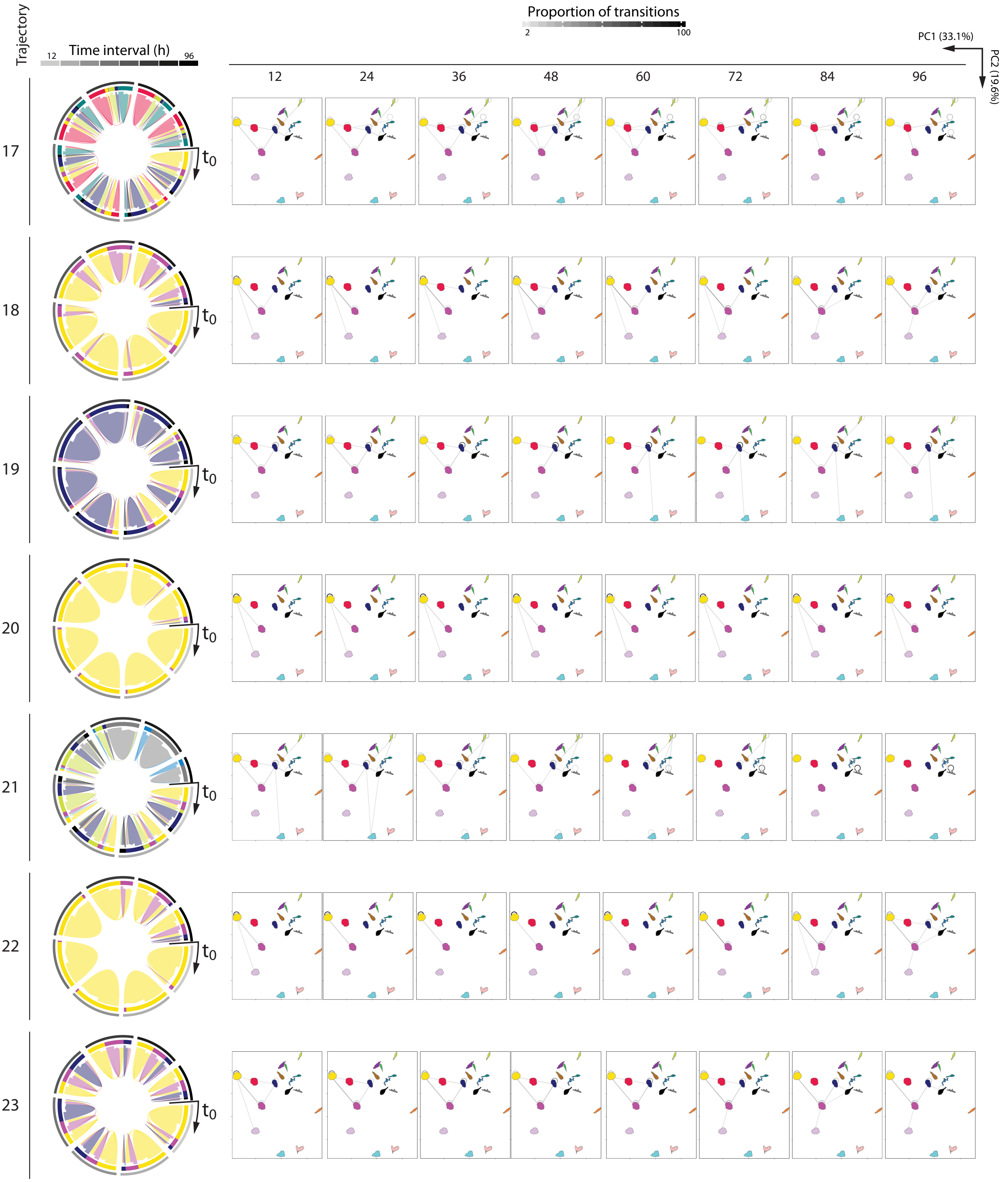

Supplementary Figure 12C

Supplementary Figure 12. Visualisation of the transitions between states within each trajectory.

A, B, and C. Visualisation of state transitions for each identified trajectory. Transitions between states shown globally as a chord diagram, with time interval in greyscale. In these diagrams, chord colour indicates the state from which the objects are transitioning. PCA was used to arrange states in 2-dimensional space; transitions (shown as proportion of total; greyscale) between, and maintaining, states are overlaid onto this for select time intervals, as straight lines or circles, respectively. Trajectories appearing in main figures – 6, 7 and 21 (Figure **3c-e**), 3 and 19 (Figure **5j and k**) and 16 and 17 (Figure **6f** and **g**) – are shown again for completeness.

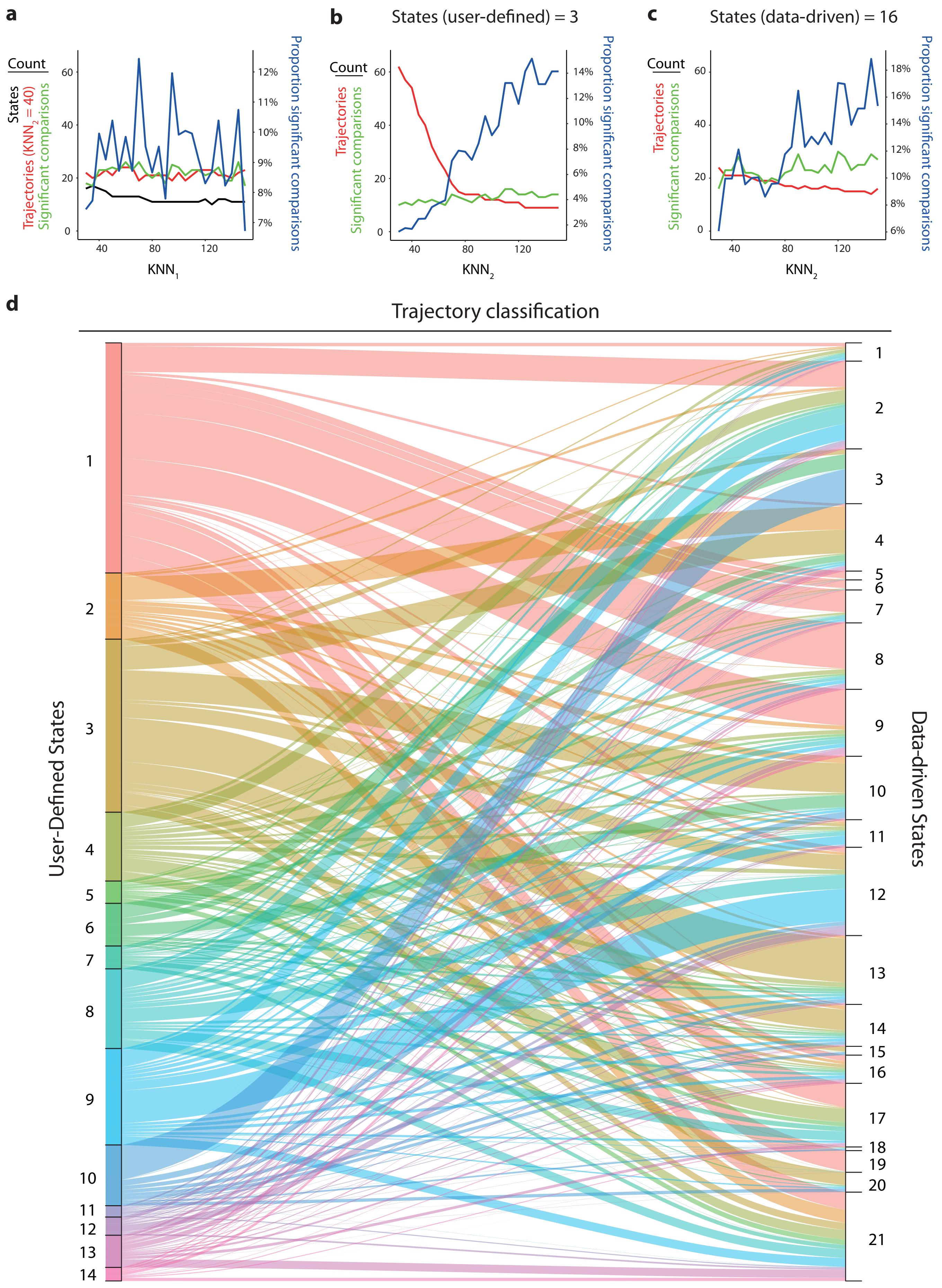

Supplementary Figure 13

Supplementary Figure 13. Testing the effect of varying k-nearest neighbour values on the resulting subpopulations.

a. Line plot showing the effect of variable *k*-nearest neighbours used by PhenoGraph to identify states (KNN_1). Resulting numbers of states and subsequent trajectories (trajectory identification performed with *k*-nearest neighbours (KNN_2) = 40) shown in black and red, respectively. Count (green) and proportion (blue) of significant enrichment/depletion of trajectories in comparison to control treatment are also shown. Comparisons were considered significant where Cochran-Mantel-Haenszel test adjusted p-value<0.05 and Woolf test adjusted p-value>0.05. Analysis performed on spheroids from all cell lines in Supplementary Table 3. Quantification of significant enrichment/depletion performed on parental PC3 and sublines, in which n=3 independent experiments, each with 3 wells/condition. Number of spheroids/condition quantified in total shown in Supplementary Table 3.

b-c. Line plot showing the effect of variable *k*-nearest neighbours used by PhenoGraph to identify trajectories (KNN_2) on the number of resulting trajectories (red), when a spheroid can be classified as one of a **(b)** few (user-defined), or **(c)** more (data-driven), states. Count and proportion of significant enrichment/depletion of trajectories in comparison to control treatment, shown in green and blue, respectively. Comparisons were considered significant where Cochran-Mantel-Haenszel test adjusted p-value<0.05 and Woolf test adjusted p-value>0.05. Analysis performed on spheroids from all cell lines in Supplementary Table 3. Quantification of significant enrichment/depletion performed on parental PC3 and sublines, in which n=3 independent experiments, each with 3 wells/condition. Number of spheroids/condition quantified in total shown in Supplementary Table 3.

d. Sankey diagram showing concordance, or lack thereof, between trajectory classifications resulting from spheroids being classified into user-defined versus data-driven states. Analysis performed on 2,532,154 spheroids from cell lines shown in Supplementary Table 3. Numbers of spheroids remaining after the filtering steps required in order to retain only well-tracked spheroids from which to determine trajectories are listed in Supplementary Table 5.

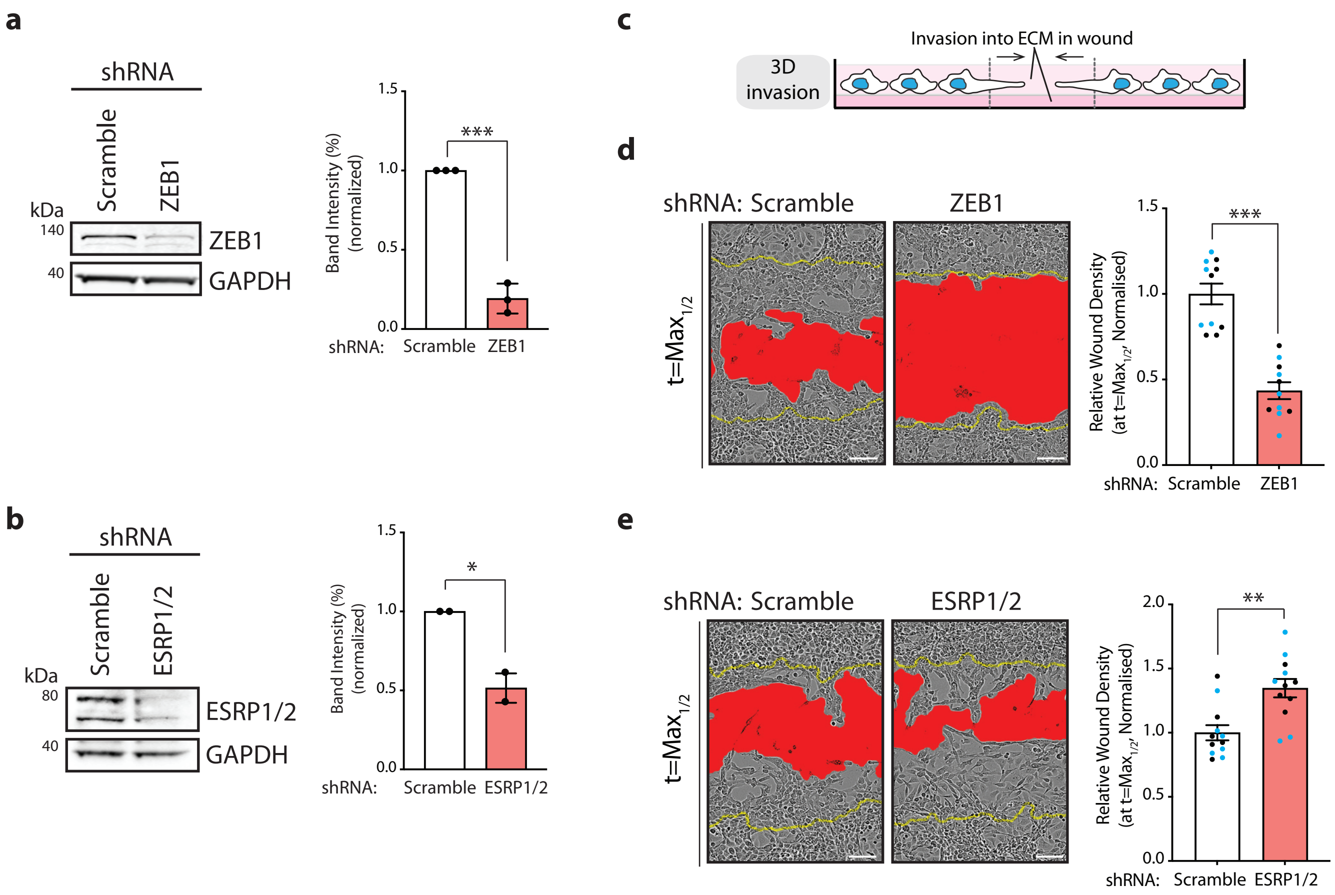

Supplementary Figure 14

Supplementary Figure 14. Knockdown of *ZEB1* or *ESRP1/2* inhibited or enhanced 3D invasion respectively.

a-b. Western blot analysis of PC3 cells stably infected with Scrambled (Scramble), **(a)** *ZEB1* or **(b)** *ESRP1/2* shRNA using anti-ZEB1, ESRP1/2 or GAPDH antibodies. GAPDH blots are the loading controls for the ZEB1 and ESRP1/2 blots. ZEB1 or ESRP1/2 intensity normalised to Scramble control was calculated. Quantitation shows values, mean ± s.d. n=3 independent experiments for *ZEB1* shRNA and n=2 independent experiments for *ESRP1/2* shRNA. p-values (Students t-test, two-tailed): *p=0.347 and ***p=0.0001**.**

c. Schema, PC3 cells plated for 24 hours before the resultant monolayers were wounded and overlaid with 25% ECM for 1 hour prior to imaging.

d-e. PC3 cells expressing Scrambled (Scramble) and **(d)** *ZEB1* or **(e)** *ESRP1/2* shRNA were plated as described in **(c)**. Representative phase contrast images are shown where the yellow dashed lines indicate initial scratch wound and red pseudo-colour shows wound. Relative wound density (RWD) at the timepoint where the Scramble controls are 50% closed ($t=Max_{1/2}$) is shown. Samples were normalised to the average of all Scramble controls across experiments. n=11 (Scramble, *ZEB1*) and 12 (Scramble, *ESRP1/2*) independent wounds generated over 2 independent experiments. Data from independent experiments is colour coded, blue and black. Values, mean ± s.d. p-values (Students t-test, two-tailed): **p≤ 0.01 and ***p ≤0.001. Scale bars, 100μm.

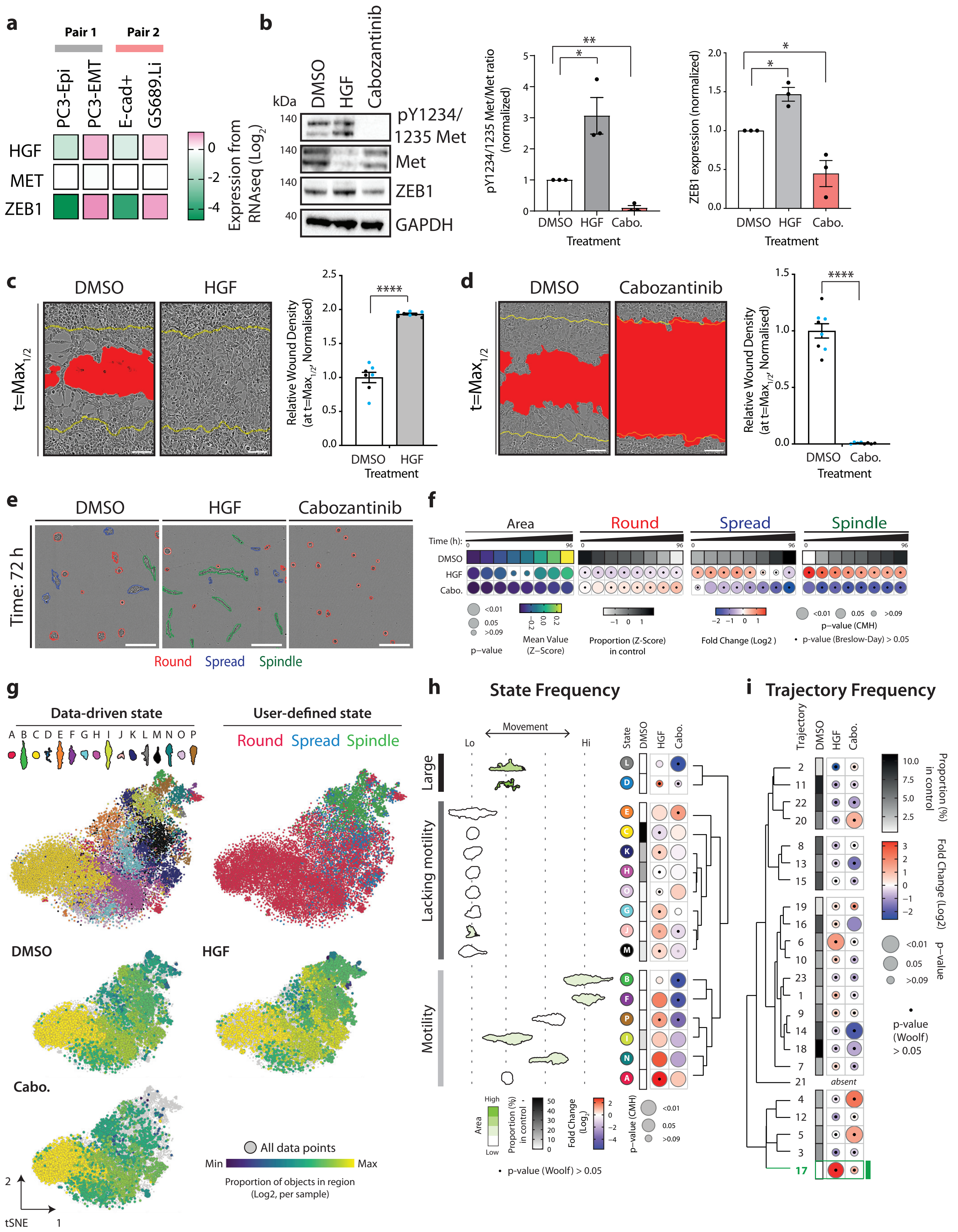

Supplementary Figure 15

Supplementary Figure 15. Manipulation of HGF signalling affects PC3 spheroid subpopulations.

a. RNAseq data comparing *HGF*, *MET* and *ZEB1* in PC3-Epi vs PC3-EMT (Pair 1) and E-Cad+ vs GS689.Li (Pair 2). Data presented as Log_2 Fold Change within each cell pair.

b. Western blot of PC3 treated with DMSO, HGF or Cabozantinib using anti-phospho Met Y1234/1235, Met, ZEB1 and GAPDH. GAPDH is loading control for phospho Met Y1234/1235 and Met and sample control for ZEB1. Representative of n=3 independent experiments. Quantification shows mean ± s.d. p-values (one-way ANOVA (ZEB): *p=0.404 (HGF) and 0.0298 (Cabo.) or Student's t-test, two-tailed (for Met)): *p=0.0248 (HGF) and **p=0.0073 (Cabo.).

c-d. Invasion assay in PC3 treated with DMSO and **(c)** HGF or **(d)** Cabozantinib. Phase images shown (yellow dashed lines, initial scratch wound and red pseudo colour, wound). RWD at the timepoint where the controls are 50% closed ($Tmax_{1/2}$) shown. Samples normalised to average of all controls across experiments. n=7 (DMSO) and 8 (HGF, DMSO and Cabozantinib) independent wounds generated over 2 independent experiments. Data from independent experiments is colour coded. Mean ± s.d. p-values (Students t-test, two-tailed): ****p< 0.0001. Scale bars, 100μm.

e. Representative phase images of PC3 spheroids, treated with DMSO, HGF or Cabozantinib, overlaid with outlines coloured by user-defined classification at 72 hours. Scale bar, 100μm.

f. Quantitation of phenotype in **(e).** Heatmaps show Area and Round, Spread or Spindle state quantitation as described in Figure **1h**. n=2 independent experiments, 4 wells/condition/experiment, quantified in Supplementary Table 3.

g. t-SNE of PC3 spheroids treated with DMSO, HGF or Cabozantinib. Plot points coloured by data-driven and user-defined state classifications. Purple-to-yellow shows per sample proportion of total objects in each data-driven state, as quantified prior to t-SNE. Spheroids quantified in Supplementary Table 3. t-SNE analysis performed on

20,000 objects subsampled via GeoSketch, with iterations; 2,000, theta; 0.5, perplexity; 50.

h. Quantitation of data-driven state classifications. Representative outlines shown and quantified as described in Figure **2e**. n described in **(f)** and spheroids quantified in Supplementary Table 3.

i. Quantitation of trajectory classifications as described in Figure **3b.** n described in **(f)** and spheroids quantified in Supplementary Table 5.

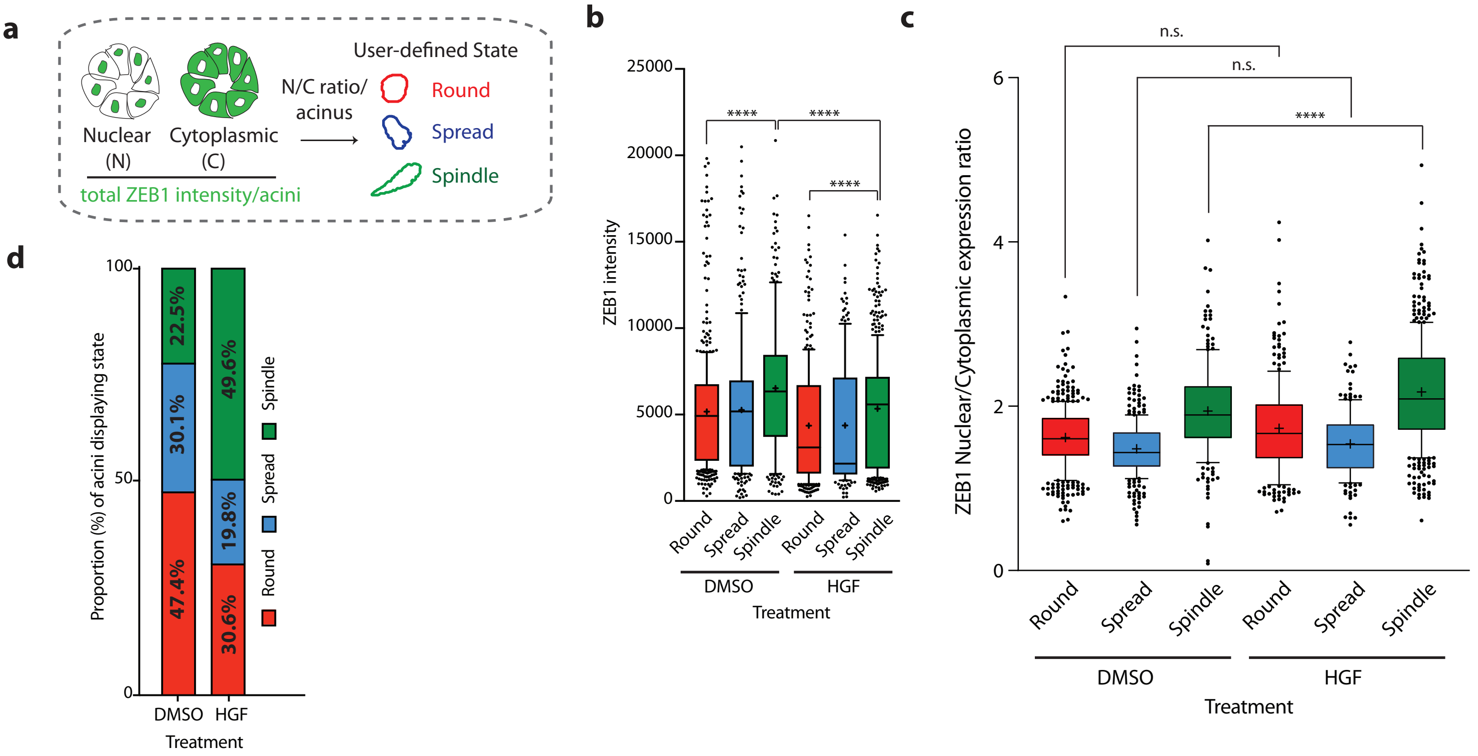

Supplementary Figure 16

Supplementary Figure 16. HGF-induced activation of Met results in increase in nuclear to cytoplasmic ratio of ZEB1 in Spindle shaped spheroids.

a. Schema, PC3 spheroids after fixing and staining with anti-ZEB1 antibody to detect nuclear (N) and cytoplasmic (C) ZEB1. A custom pipeline was used to quantify the N:C ratio of ZEB1 intensity and user-defined state classification (Round, Spindle or Spread) of each spheroid.

b-c. After 2 days, PC3 spheroids were treated with either DMSO or HGF for 24 hours, fixed and then stained with anti-ZEB1 antibody, Hoechst (nucleus) and HCS CellMask Deep Red Stain (whole cell). Spheroids were imaged using a Phenix Opera and the user-defined state and ZEB1 intensity in the nucleus and the cytoplasm measured for each spheroid using a custom pipeline in Harmony High-Content Imaging and Analysis Software (PerkinElmer, Version 4.6). **(b)** Box and whiskers plot shows total ZEB1 intensity/spheroid: 10–90 percentile; +, mean; dots, outliers; midline, median; boundaries, quartiles. p-values (One-Way ANOVA): ****$p \leq 0.0001$. **(c)** N:C ratio for spheroids of each state were calculated and data are presented in a box and whiskers plot: 10–90 percentile; +, mean; dots, outliers; midline, median; boundaries, quartiles. p-values (Students t-test, two-tailed): ****$p \leq 0.0001$. n=2 experimental replicates, each with 4 wells/condition. 875 (HGF) and 965 (DMSO) spheroids quantified in total. Note increase in nuclear ratio of ZEB1 in Spindle state spheroids.

d. Proportion of spheroids displaying each user-defined state (Round, Spread or Spindle) in **(b-c)** is shown for each condition. Note increase in Spindle state spheroids in response to HGF.

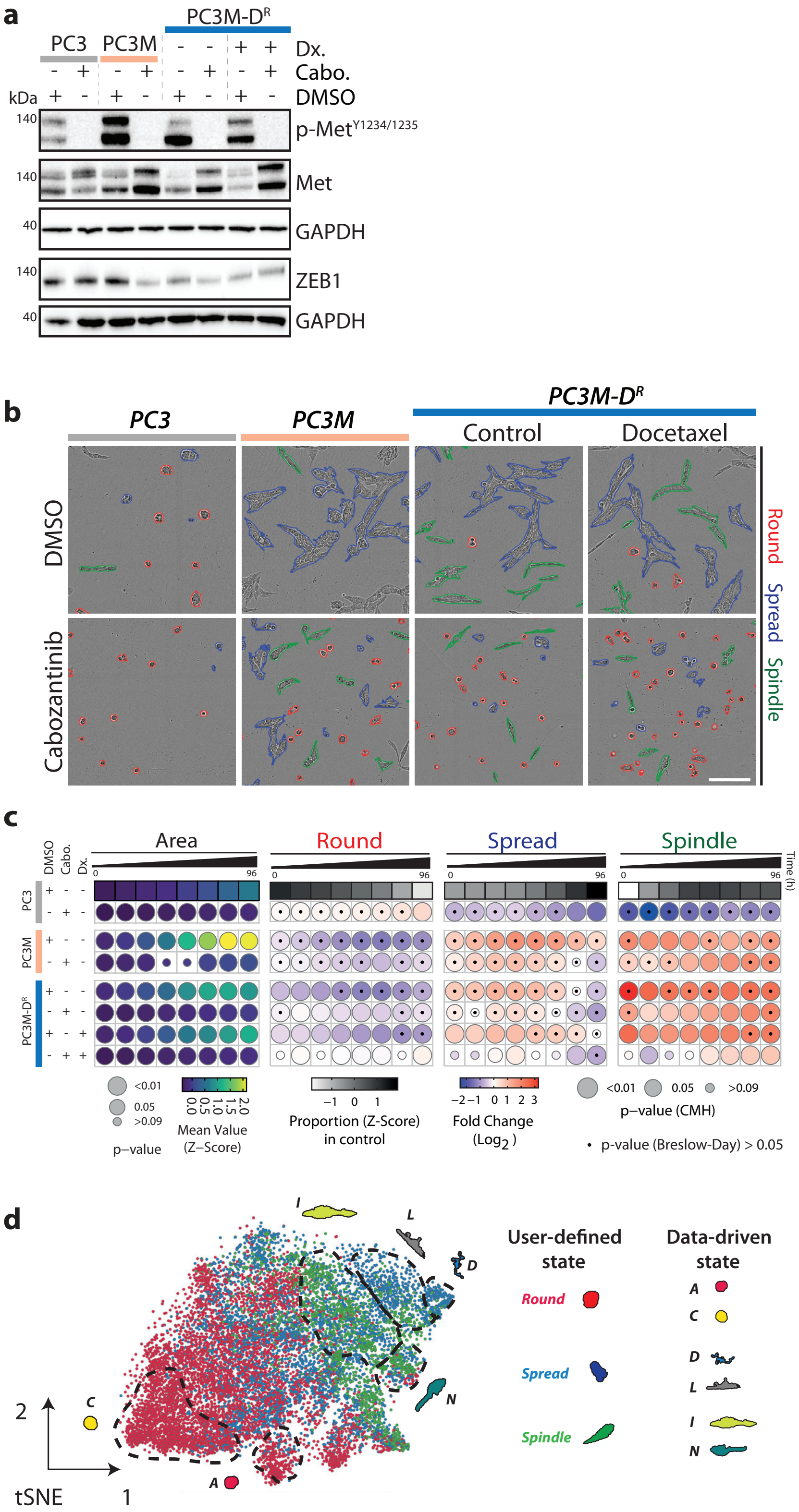
a
PC3
PC3M
PC3M-D[R]
Dx.
Cabo.
DMSO
kDa
p-Met[Y1234/1235]
Met
GAPDH
ZEB1
GAPDH
b
PC3
PC3M
PC3M-D[R]
Control
Docetaxel
DMSO
Cabozantinib
Round
Spread
Spindle
c
Area
Round
Spread
Spindle
Time (h)
p-value
Mean Value (Z-Score)
Proportion (Z-Score) in control
Fold Change (Log2)
p-value (CMH)
p-value (Breslow-Day) > 0.05
d
User-defined state
Data-driven state
Round
Spread
Spindle
tSNE

Supplementary Figure 17. Application of user-defined classifications to a metastasis and drug resistance derivative series of PC3 spheroids.

a. Western blot of PC3, PC3M and PC3M-D^R treated with DMSO or Cabozantinib (Cabo.) and PC3M-D^R treated with Docetaxel (Dx.) using anti-phospho Met Y1234/1235, Met, ZEB1 and GAPDH antibodies. Upper and lower GAPDH blots are the loading control for pMet/Met and for ZEB1 blots, respectively. Representative of n=3 independent experiments.

b. Phase images of PC3, PC3M and PC3M-D^R spheroids treated with DMSO, Cabozantinib and/or Docetaxel overlaid with user-defined state classification outlines at 72 hours. Representative of n=2 independent experiments, each with 4 wells/condition. Scale bar, 100μm.

c. Quantitation of phenotypes exemplified in **(b)**. Heatmaps show Area measurement as mean of Z-score normalised values (purple to yellow), and classification of spheroids into Round, Spread or Spindle as a Log_2 Fold Change from control (PC3 + DMSO) (blue to red). Proportion of control at each timepoint is also Z-score normalised for each class (white to black). p-values, Student's t-test (two-sided) and Cochran-Mantel-Haenszel test, both Bonferroni adjusted, to compare area and proportion of each classification to control respectively, represented by bubble size. p-value, Breslow-Day test, Bonferroni-adjusted for homogeneity of odds ratio across experimental replicates, represented by dot. n=2 independent experiments, each with 4 wells/condition. Spheroids quantified in Supplementary Table 3.

d. t-SNE visualisation of PC3, PC3M and PC3M-D^R spheroids treated with Cabozantinib and/or Docetaxel from Figure **6b, c**. Plot points coloured by user-defined state classifications. Black dashed lines were manually annotated to highlight regions corresponding to data-driven states discussed in text. Total spheroids/condition listed in Supplementary Table 3. t-SNE analysis performed on 20,000 objects subsampled via GeoSketch, with iterations; 5,000, theta; 0.25, perplexity;100. Note that whereas Round and Spread states are highly concordant with data-driven classifications A, C and D, L, respectively, states I, N overlap with Spindle state but also contain high

levels of Spread state. This is an indication of why both Spread and Spindle states are seemingly induced in PC3M and PC3M-D^R.

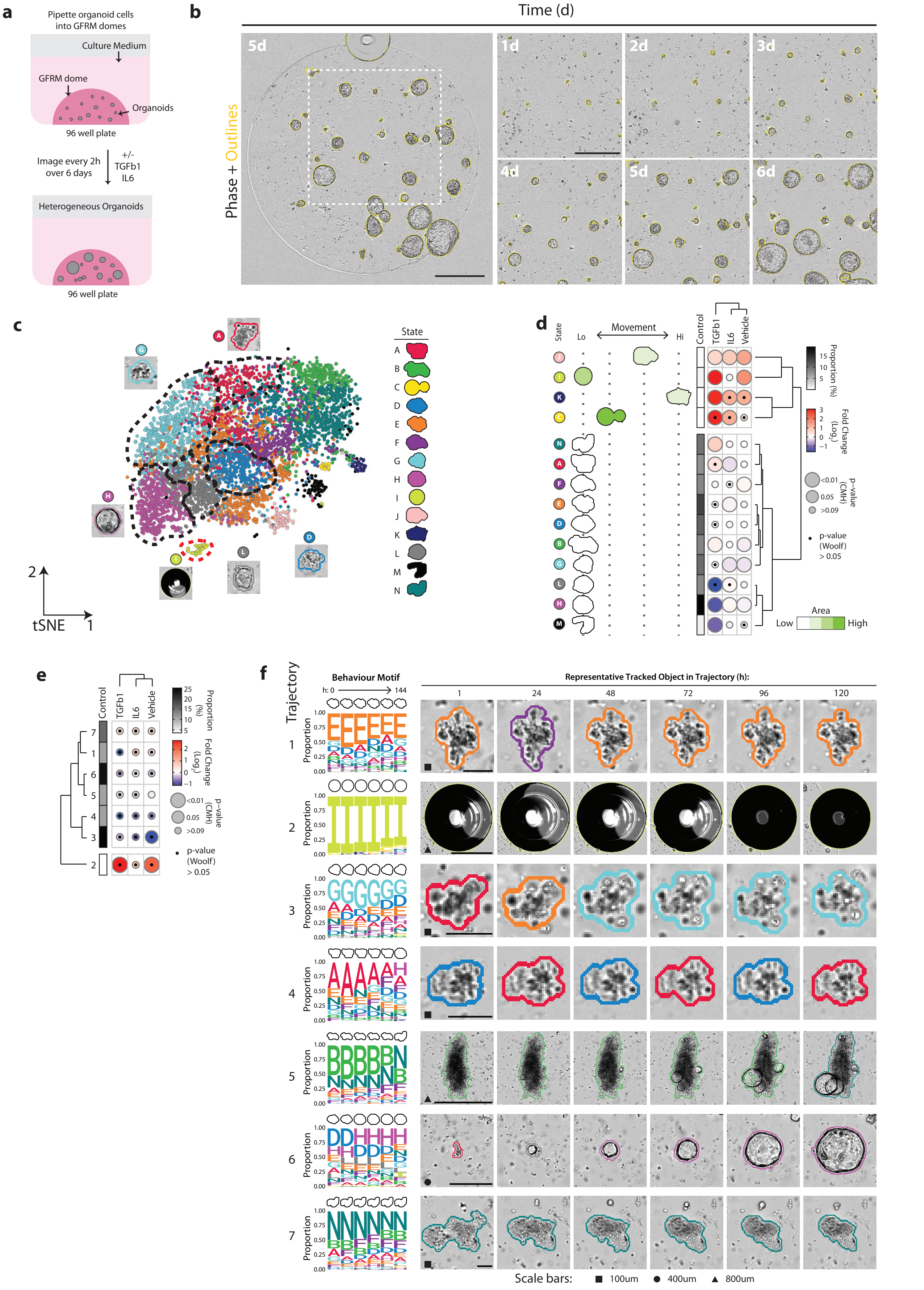

Supplementary Figure 18

Supplementary Figure 18. Identification of states and trajectories in live-imaging of murine organoids cultured in domes.

a. Cartoon depicting murine organoids cultured in domes of GFRM for imaging using the IncuCyte® S3 IncuCyte Organoid Analysis Software Module.

b. Representative phase image and zoomed images of boxed region shown for *villin*Cre^{ER}; *Kras*$^{G12D/+}$; *Trp53*$^{fl/fl}$; *Rosa26*$^{N1icd/+}$ organoid line RBVKPN RKAC13.1g. Object outlines, yellow. n=3 independent experiments, 2-3 wells/condition/experiment. Scale bars, 800µm.

c. t-SNE of objects from KPN organoids, untreated or treated with vehicle, TGFβ1 or IL6. Plot points coloured by data-driven state classification. Black dashed lines highlight regions corresponding to data-driven states. Data comprised of each object identified in each image frame of the experiment. Total object counts in Supplementary Table 6. t-SNE analysis performed on 20,000 objects subsampled via GeoSketch, with iterations; 5,000, theta; 0.25, perplexity; 50. Brightfield images with outlines pseudo-coloured by state classification provided.

d. Quantitation of data-driven state classifications. Representative outlines shown and quantified as described in Figure **2e**. n=4 experimental replicates with 2-3 wells/condition. Total organoids quantified in Supplementary Table 6.

e. Quantitation of trajectory classifications as described in Figure **3b.** The total objects/condition quantified after filtering steps, are listed in Supplementary Table 6.

f. Trajectory visualisation. Colours correspond to the states identified previously and shown in **(c)**. Behaviour motif depicting frequency (proportion) of states in 24-hour time intervals, with outline of the most abundant state shown at top. Using the most frequent state at each timepoint, an object was selected to represent the trajectory. Brightfield images of these are shown, with outline colour indicating state classification at given timepoint. Scale bars, 100µm (square), 400µm (circle), and 800µm (triangle).

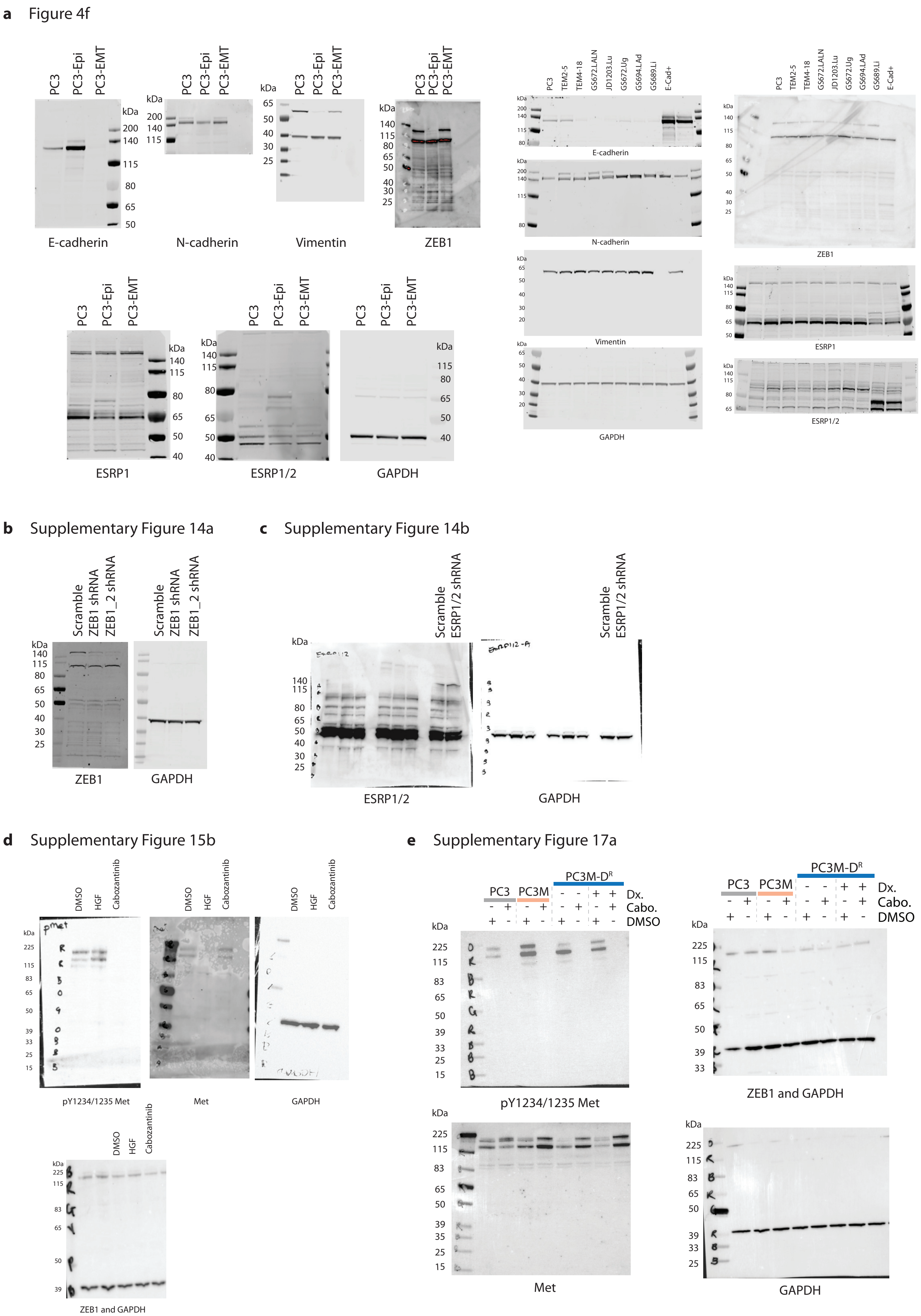

Supplementary Figure 19

Supplementary Figure 19. Raw images of western blot data.

a-e. Uncropped and unprocessed images of western blots used throughout manuscript are shown.

Supplementary Table 1

Glossary

**2D**	2-Dimensional culture. The culturing of cells in conditions that provide two axes of orientation, the rigid substratum and the overlaid medium.
**3D**	3-Dimensional culture. The embedding of cells in extracellular matrix or hydrogels such that the orientation of the axes of polarity is generated by the cells, rather than the culture dish.
**4D**	The analysis of 3D over time.
**Spheroid**	A collection of epithelial cells forming a 3D structure, which may include surrounding a lumen.
**Object**	The region of an image that is detected by image segmentation. This is usually a single cell at early timepoint and becomes the collection of cells that together comprise an acinus over time.
**Feature**	A variable that is measured from an object.
**State**	An object class, either user-defined or data-driven. States are defined by a combination of features that are measured from objects in the dataset. Every object identified in every image is classified into a state, and as a consequence the time interval this covers is determined by the imaging frequency.
**Cell state**	A combination of features that define a cell.
**Classifier**	A collection of features used in a machine learning approach to identify a distinct state.
**State space**	The repertoire of states and their relation, defined by their underpinning features, to each other.
**Morphogenesis**	The development of a phenotype over time.
**Trajectory**	The sequence of states during morphogenesis over time.

Supplementary Table 1

Glossary of terms used.

Supplementary Table 2

Figure	Sample	Experiment	Replicate Count	Acini Count	Total Acini/Condition
Supplementary Figures 1 & 2	PC3	1	3	28403	112476
		2	3	52239	
		3	3	31834	
	RWPE-1	1	4	59709	132876
		2	4	19255	
		3	4	53912	
	RWPE-2	1	4	43629	105875
		2	4	33298	
		3	4	28948	
	CWR	1	4	51408	120214
		2	4	68806	
	22Rv1	1	4	61370	113878
		2	4	52508	
	PC3M	1	4	25686	63518
		2	4	37832	
	PC3M-DR	1	4	38350	73478
		2	4	35128	
	Caco-2	1	4	23198	74756
		2	4	27757	
		3	4	23801	
	MDA-MB-231	1	4	23417	55458
		2	4	18694	
		3	4	13347	
	MDCK	1	3	21023	21023
Supplementary Figures 1 & 3	KC_1	1	4	15887	50766
		2	3	15098	
		3	4	19781	
	KC_2	1	4	26104	61525
		2	3	15485	
		3	4	19936	
	KC_3	1	4	22041	65315
		2	6	28932	
		3	4	14342	
	KPC_1	1	4	28896	67049
		2	3	17127	
		3	4	21026	
	KPC_2	1	4	24059	72709
		2	6	28102	
		3	4	20548	
	KPC_3	1	4	31781	85515
		2	3	26201	
		3	4	27533	
	KPFLC_1	1	4	18135	61284
		2	3	20252	
		3	4	22897	
	KPFLC_2	1	4	23041	85385
		2	6	38084	
		3	4	24260	
	KPFLC_3	1	4	24838	64120
		2	3	17206	
		3	4	22076	
	Pten_2	1	4	9220	39715
		2	3	14949	
		3	4	15546	
	Pten_3	1	4	13662	67208
		2	6	35993	
		3	4	17553	
	Pten_4	1	4	25150	68441
		2	6	25355	
		3	4	17936	

Supplementary Table 2

Summary of numbers of experimental and technical replicates, and spheroids imaged per condition in the preliminary analysis of 22 cell lines representing different tissues of origin or multiple independent lines derived from the same mouse tumour genotypes.

Supplementary Table 3

Figure	Sample	Experiment	Replicate Count	Acini Count	Total Acini/Condition
Figures 1-2, Supplementary Figures 4, 6-8, 13	PC3	1	3	33942	126955
		2	3	55742	
		3	3	37271	
	PC3-Epi	1	3	43718	115438
		2	3	38630	
		3	3	33090	
	PC3-EMT	1	3	22153	76127
		2	3	31520	
		3	3	22454	
	E-cad+	1	3	45474	130029
		2	3	51562	
		3	3	32993	
	GS689.Li	1	3	35472	111706
		2	3	44585	
		3	3	31649	
	GS672.Ug	1	3	42399	118479
		2	3	51321	
		3	3	24759	
	GS694.LAd	1	3	53427	136465
		2	3	52447	
		3	3	30591	
	TEM2-5	1	3	36343	132918
		2	3	43729	
		3	3	52846	
	TEM4-18	1	3	40106	99968
		2	3	39622	
		3	3	20240	
	JD1203.Lu	1	3	40253	123706
		2	3	62507	
		3	3	20946	
	GS683.LALN	1	3	51414	108995
		2	3	40316	
		3	3	17265	
Figure 5, Supplementary Figures 6, 8	Scramble ZEB1	1	4	51280	91770
		2	4	40490	
	ZEB1	1	4	39169	66238
		2	4	27069	
	ZEB1_2	1	4	63002	116857
		2	4	53855	
	Scramble ESRP	1	4	51756	197741
		2	4	80935	
		3	4	65050	
	GIP Scramble	1	4	37617	180351
		2	4	69187	
		3	4	73547	
	RIB Scramble	1	4	86072	230142
		2	4	75775	
		3	4	68295	
	ESRP1	1	4	71211	174889
		2	4	55640	
		3	4	48038	
	ESRP2	1	4	56016	164788
		2	4	61358	
		3	4	47414	
	ESRP1/2	1	4	32737	160102
		2	4	79114	
		3	4	48251	
Supplementary Figures 6, 8,15	PC3 + DMSO	1	3	14583	35225
		2	3	20642	
	PC3 + HGF	1	3	20299	43619
		2	3	23320	
	PC3 + Cabozantinib	1	3	17256	41122
		2	3	23866	
Figure 6, Supplementary Figures 6, 8, 17	PC3 + DMSO	1	4	29747	60934
		2	4	31187	
	PC3 + Cabozantinib	1	4	39559	83532
		2	4	43973	
	PC3M + DMSO	1	4	31250	61390
		2	4	30140	
	PC3M + Cabozantinib	1	4	65517	121299
		2	4	55782	
	PC3MDR + DMSO	1	4	30139	55398
		2	4	25259	
	PC3MDR + Cabozantinib	1	4	49440	97246
		2	4	47806	

Supplementary Table 3

	PC3MDR + Docetaxal	1	4	31211	56910
		2	4	25699	
	PC3MDR + Cabo. + Doc.	1	4	64020	78842
		2	4	14822	

Supplementary Table 3

Summary of numbers of experimental and technical replicates, and spheroids imaged per condition in the analysis of parental PC3 and sublines.

Supplementary Table 4

**Type**	**Name**	
Size	Area	
Shape	Zernike	0_0
		1_1
		2_0
		2_2
		3_1
		3_3
		4_0
		4_2
		4_4
		5_1
		5_3
		5_5
		6_0
		6_2
		6_4
		6_6
		7_1
		7_3
		7_5
		7_7
		8_0
		8_2
		8_4
		8_6
		8_8
		9_1
		9_3
		9_5
		9_7
		9_9
Movement	Displacement	
	DistanceTraveled	

Supplementary Table 4

List of non-redundant size, shape, and motility features measured by CellProfiler, which were used by Traject3d.

Supplementary Table 5

Figure	Sample	Experiment	Replicate Count	Acini Count	Total Acini/Condition	Total Tracked/Condition
Figures 3, Supplementary Figures 10-12	PC3	1	3	22709	62389	695
		2	3	19666		
		3	3	20014		
	PC3-Epi	1	3	23688	58641	637
		2	3	20219		
		3	3	14734		
	PC3-EMT	1	3	11671	31438	349
		2	3	7650		
		3	3	12117		
	E-cad+	1	3	26706	72904	786
		2	3	27453		
		3	3	18745		
	GS689.Li	1	3	20978	58951	634
		2	3	19821		
		3	3	18152		
	GS672.Ug	1	3	25034	68986	773
		2	3	25519		
		3	3	18433		
	GS694.LAd	1	3	21194	63121	704
		2	3	25045		
		3	3	16882		
	TEM2-5	1	3	17909	59301	661
		2	3	25389		
		3	3	16003		
	TEM4-18	1	3	3322	29516	333
		2	3	12631		
		3	3	13563		
	JD1203.Lu	1	3	30270	86513	916
		2	3	42480		
		3	3	13763		
	GS683.LALN	1	3	25229	61359	660
		2	3	24077		
		3	3	12053		
Figure 5, Supplementary Figures 10-12	Scramble ZEB1	1	4	31451	56815	609
		2	4	25364		
	ZEB1	1	4	29722	47550	501
		2	4	17828		
	ZEB1_2	1	4	40077	74296	789
		2	4	34219		
	Scramble ESRP	2	4	52857	97405	1077
		3	4	44548		
	GIP Scramble	2	4	52323	95450	1014
		3	4	43127		
	RIB Scramble	1	1	1610	91842	980
		2	4	41482		
		3	4	48750		
	ESRP1	2	4	41869	77984	827
		3	4	36115		
	ESRP2	1	2	1391	77301	820
		2	4	41752		
		3	4	34158		
	ESRP1/2	2	4	47218	81876	897
		3	4	34658		
Supplementary Figures 10-12, 15	PC3 + DMSO	1	3	8506	21674	230
		2	3	13168		
	PC3 + HGF	1	3	4232	10825	115
		2	3	6593		
	PC3 + Cabozantinib	1	3	13816	29504	310
		2	3	15688		
Figure 6, Supplementary Figures 10-12, 17	PC3 + DMSO	1	4	20844	41937	447
		2	4	21093		
	PC3 + Cabozantinib	1	4	28778	60795	639
		2	4	32017		
	PC3M + DMSO	1	4	9101	17451	201
		2	4	8350		
	PC3M + Cabozantinib	1	4	35840	68924	737
		2	4	33084		
	PC3MDR + DMSO	1	4	9870	15508	175
		2	4	5638		
	PC3MDR + Cabozantinib	1	4	28163	59783	633
		2	4	31620		
	PC3MDR + Docetaxal	1	4	6032	21875	244
		2	4	15843		
	PC3MDR + Cabo. + Doc.	1	4	39269	50133	529
		2	4	10864		

Supplementary Table 5

Summary of numbers of experimental and technical replicates, and spheroids imaged per condition, in the analysis of parental PC3 and sublines after filtering to retain only spheroids which were tracked sufficiently over the course of the experiment.

Supplementary Table 6

Sample	Experiment	Replicate Count	Object Count	Total Objects /Condition	Trajectory Object Count	Total Trajectory Objects/Condition	Tracked Object Count
TGFβ1	1	2	2878	11907	2878	11907	2334
	2	3	3191		3191		
	3	3	3283		3283		
	4	3	2555		2555		
IL-6	1	3	9346	31064	9346	31064	3315
	2	2	4223		4223		
	3	3	8604		8604		
	4	3	8891		8891		
Control	1	3	8434	28983	8434	28983	2973
	2	3	5430		5430		
	3	3	6527		6527		
	4	3	8592		8592		
Vehicle	1	3	7237	25743	7237	25743	2673
	2	3	4965		4965		
	3	3	6523		6523		
	4	3	7018		7018		

Supplementary Table 6

Summary of numbers of experimental and technical replicates, and objects imaged per condition, in the analysis of murine organoids. Object counts shown before (“Object Count”, “Total Objects/Condition”), and after (“Trajectory Object Count”, “Total Trajectory Objects/Condition”), filtering to retain only those which were tracked sufficiently over the course of the experiment. “Tracked Object Count” represents the number of tracked objects classified into trajectories.

Supplementary Table 7

Target Gene	shRNA target sequence (5' – 3')
shScr	CCGCAGGTATGCACGCGT
shZeb1	AACAATACAAGAGGTTAAACTC
shESRP1	CACAATGACAGAGUATTTAAA
shESRP2	AGCCCGAGGTGATAAAGC

Supplementary Table 7

List of shRNA target sequences.

Supplementary Note 1

Altering the number of other objects each object is compared to during definition of behaviour states (*k*-Nearest Neighbour, KNN_1) revealed a modest inverse relationship between KNN_1 and the number of behaviour states identified, but that the number and proportion of resulting trajectories was largely stable (Supplementary Figure 13a). When using three broad user-defined behaviour states, altering KNN_2 (the analogous function to find trajectories, rather than states) resulted in a bi-phasic response in the number of trajectories and therefore the proportion that display significance, but still had a modest effect on the number of significant trajectories (Supplementary Figure 13b). In contrast, altering KNN_2 when using complex state definitions (16 states) also resulted in a largely stable number of trajectories, but gave a much less dramatic total/proposition significant trajectory ratio (Supplementary Figure 13c). Crucially, the absolute number of significant trajectories was 1.5-fold higher when behaviour states were defined using granular, automated subtype analysis than broad, user-defined classifiers (3 classes), across all KNN alterations. This did not represent simple subdivision of broad subtype trajectories, but rather distinct new trajectories not easily identified by a limited repertoire of behaviour states (Supplementary Figure 13d). This suggests that increased computational cost of increased KNN_1 or KNN_2 does not stably enhance identification of significant morphogenesis trajectories, and that data-driven identification of distinct behaviour states is superior to simple, broad classifications in identifying temporal morphogenesis patterns.
