# Supplementary material for: Traject3d allows label-free identification of distinct co-occurring phenotypes within 3D culture by live imaging
Source: Nat Commun. 2022 Sep 9;13:5317. doi: 10.1038/s41467-022-32958-x (PMC9463449; doi:10.1038/s41467-022-32958-x)
Supplement: Supplementary file 2 — Reporting Summary [file 41467_2022_32958_MOESM2_ESM.pdf]

nature research

Corresponding author(s): David Bryant

Last updated by author(s): Aug 15, 2022

Reporting Summary

Nature Research wishes to improve the reproducibility of the work that we publish. This form provides structure for consistency and transparency in reporting. For further information on Nature Research policies, see our Editorial Policies and the Editorial Policy Checklist.

Statistics

For all statistical analyses, confirm that the following items are present in the figure legend, table legend, main text, or Methods section.

n/a	Confirmed	
☐	☒	The exact sample size (*n*) for each experimental group/condition, given as a discrete number and unit of measurement
☐	☒	A statement on whether measurements were taken from distinct samples or whether the same sample was measured repeatedly
☐	☒	The statistical test(s) used AND whether they are one- or two-sided<br>*Only common tests should be described solely by name; describe more complex techniques in the Methods section.*
☐	☒	A description of all covariates tested
☐	☒	A description of any assumptions or corrections, such as tests of normality and adjustment for multiple comparisons
☐	☒	A full description of the statistical parameters including central tendency (e.g. means) or other basic estimates (e.g. regression coefficient) AND variation (e.g. standard deviation) or associated estimates of uncertainty (e.g. confidence intervals)
☐	☒	For null hypothesis testing, the test statistic (e.g. *F*, *t*, *r*) with confidence intervals, effect sizes, degrees of freedom and *P* value noted<br>*Give P values as exact values whenever suitable.*
☒	☐	For Bayesian analysis, information on the choice of priors and Markov chain Monte Carlo settings
☒	☐	For hierarchical and complex designs, identification of the appropriate level for tests and full reporting of outcomes
☐	☒	Estimates of effect sizes (e.g. Cohen's *d*, Pearson's *r*), indicating how they were calculated

Our web collection on statistics for biologists contains articles on many of the points above.

Software and code

Policy information about availability of computer code

Data collection

Data from real-time imaging of 3D acini and invasion assays was collected using Incucyte ZOOM Live Cell Analysis System Software 2018A (Essen Biosciences) or Incucyte S3 Live-Cell Analysis System (2021A).

For high throughput image collection of 3D acini cultures we utilized an Opera Phenix Z9501 High-Content Imaging System with the associated software package Harmony High-Content Imaging and Analysis Software (PerkinElmer, Version 4.6).

Images from western blots were collected using a ChemiDoc Imager (BioRad) using Image Lab 6.1 software or an Odyssey Imaging System (LI-COR Biosciences) using Image Studio Software 6.0.

Data analysis

Incucyte ZOOM Live Cell Analysis System software (Essen Biosciences, 2018A) was used to analyze the Relative Wound Density (RWD) in invasion assays and Incucyte Organoid Analysis Software Module used to generate outlines of objects.

Training for user-defined phenotype classifications was performed in CellProfiler Analyst (v2.2.0). A custom pipeline in CellProfiler (v3.1.8) was used to generate outlines of phase acini, track acini between frames, and generate measurements of size, shape, and movement.

RNAseq data was analysed using DESeq2 (v1.18.1) and Metacore (https://portal.genego.com, November 2017 version).

A custom pipeline designed in KNIME Data Analytics Platform (v4.0.2) was used to collate data (generated by CellProfiler) from multiple experiments, parse user-defined classifications, and calculate Z-score normalisation. R (v4.2.0) and Python (v3.8) KNIME integrations were used for subsequent analysis in this pipeline. Generally analysis in this pipeline was performed using base KNIME functionality coupled with custom R and Python scripts which utilise existing packages. A custom R script was used to correct the duplicated tracking ID generated for acini by CellProfiler. Subsampling was performed using the GeoSketch (v1.0) Python package. Behaviour states were identified using the PhenoGraph algorithm, as implemented in the cytofkit2 R package. A custom R script was used for selecting a representative object for each

behaviour state classification. Trajectories were identified using imputeTS (v3.0) to impute missing values, and FactoMineR (v2.0) to convert categorical variables to numeric values, before clustering the data using PhenoGraph. tSNE visualisation was performed using the Rtsne (v0.15) package. Woolf and Breslow-Day tests were performed using the vcd (v1.4-4) and DescTools (v0.99.31) R packages, respectively. All plots were generated using the ggplot2 (v 3.3.0) R package, apart from the heatmap of acini behaviour state sequences, trajectory behaviour state motifs, and behaviour state transition chord diagrams, which were generated using the pheatmap (v1.0.12), ggseqlogo (v0.1), and circlize (v0.4.10) R packages, respectively. The custom KNIME pipeline, including all R and Python scripts, custom and otherwise, which utilise the packages described above will be available on the Traject3d GitHub repository (https://github.com/davebryantlab/Traject3d). We have updated our pipeline (provided on GitHub) to utilise current versions of R (v4.2.0) and the above packages, all of which are listed in the associated user manual.

UMAP was performed independently of the KNIME pipeline described above, using the umap (v0.2.4.0) R package.

Harmony High-Content Imaging and Analysis Software (PerkinElmer, Version 4.6) was used to analyze fixed 3D PC3 acini stained with anti-ZEB1 antibody, Hoechst and HCS CellMask. Used in combination these dyes allowed the generation of a nuclear mask (all nuclei) and a cytoplasmic mask (whole acini excluding all nuclei) for each acinus. Total intensity of anti-ZEB1 staining was then measured in the nuclei (N) and in the cytoplasmic region (C) of each acinus. The morphological properties of each acinus were calculated to classify them into three subpopulations (Round, Spread and Spindle) using machine learning following manual training. Prism 9 was used to present data in box and whiskers plots as nuclear to cytoplasmic ratio (N:C) of total ZEB1 intensity or total ZEB1 intensity for each acini/subpopulation/treatment. The proportion of each acini subpopulation (Round, Spread, Spindle) is also presented.

Quantitation of western blots was carried out using Image Lab 6.1 software (BioRad) or Image Studio Software 6.0. (LI-COR Biosciences).

For manuscripts utilizing custom algorithms or software that are central to the research but not yet described in published literature, software must be made available to editors and reviewers. We strongly encourage code deposition in a community repository (e.g. GitHub). See the Nature Research guidelines for submitting code & software for further information.

Data

Policy information about availability of data

All manuscripts must include a data availability statement. This statement should provide the following information, where applicable:

- Accession codes, unique identifiers, or web links for publicly available datasets
- A list of figures that have associated raw data
- A description of any restrictions on data availability

Data Availability
The RNAseq data from PC3 sublines used in this study are available in either the Short Read Archive database for PC3 E-cad+, GS689.Li in SRS354082 [https://www.ncbi.nlm.nih.gov/sra/?term=SRS354082]), or the Gene Expression Omnibus for PC3-Epi, PC3-EMT14 in GSE48230 [https://www.ncbi.nlm.nih.gov/geo/query/acc.cgi?acc=GSE48230]. Unprocessed and uncropped western blot images are provided in Supplementary Figure 19 and as Source Data.

Source Data for all Figures and Supplementary Figures are provided with this paper. Any other data supporting the findings of this study are available from the corresponding author upon reasonable request.

Code availability
All CellProfiler and KNIME analysis workflows, as well as a download link for sample data can be found in the Traject3d GitHub repository (https://github.com/davebryantlab/Traject3d).

Field-specific reporting

Please select the one below that is the best fit for your research. If you are not sure, read the appropriate sections before making your selection.

☒ Life sciences ☐ Behavioural & social sciences ☐ Ecological, evolutionary & environmental sciences

For a reference copy of the document with all sections, see nature.com/documents/nr-reporting-summary-flat.pdf

Life sciences study design

All studies must disclose on these points even when the disclosure is negative.

Sample size	Our studies involve growth of cells as 3D cysts, often in a 96-well plate to allow for high-throughput imaging. Germaine to this is plating of cysts with appropriate density (neither too dense or sparse) to facilitate optimal growth without overcrowding, the latter of which causes aberrant merging of cysts. In this format, we can image hundreds to thousands of spheroids per condition. Such high samples numbers preclude the need to pre-calculate minimal sample size to achieve significant effects, allowing robust statistical depth.
Data exclusions	No data were excluded in analyses.
Replication	Where appropriate number of biological and technical replicates are stated in Figure Legends and Supplementary Tables 2,3, and 5.

Randomization	Due to the nature of this research, randomization is not appropriate as assignment to categories must be exact for the data analysis workflow to function. Due to the automated nature of our analysis pipelines users have no control of raw values generated from experiments.
Blinding	Blinding was not performed. However, experimenter bias was circumvented by the use of automated imaging and data analysis pipelines.

Reporting for specific materials, systems and methods

We require information from authors about some types of materials, experimental systems and methods used in many studies. Here, indicate whether each material, system or method listed is relevant to your study. If you are not sure if a list item applies to your research, read the appropriate section before selecting a response.

Materials & experimental systems

n/a	Involved in the study	
☐	☒	Antibodies
☐	☒	Eukaryotic cell lines
☒	☐	Palaeontology and archaeology
☒	☐	Animals and other organisms
☒	☐	Human research participants
☒	☐	Clinical data
☒	☐	Dual use research of concern

Methods

n/a	Involved in the study	
☒	☐	ChIP-seq
☒	☐	Flow cytometry
☒	☐	MRI-based neuroimaging

Antibodies

Antibodies used	Western blots - all 1:1,000, unless stated otherwise: anti-GAPDH (14C10) (CST 2118 (1:5,000)), anti-Met (25H2) (CST 3127), anti-Met phospho 1234/1235 (D26) (CST 3077), anti-N-cadherin (D4R1H) (CST 13116), anti-vimentin (V9) (Santa Cruz, sc-6260), anti-E-cadherin (Clone 36) (BD Biosciences 610181), anti-ESRP1 (210-301-B89S) and anti-ESRP1/2 (210-301-C31S) (both Tebu-bio) and anti-ZEB1 (Sigma HPA027524).<br><br>Immunofluorescence - anti-ZEB1 antibody (Sigma, HPA027524) added overnight (1:100) at 4°C. Alexa Fluoro 568 Phalloidin (1:200, A12380), Alexa Fluor 488 Donkey anti-rabbit secondary antibody (1:200, A21206), HCS CellMask™ Deep Red Stain (1:50000, H32721) and Hoechst 34580 (1:1000, H21486) (all Thermo Fisher Scientific).
Validation	Anti-ZEB1 (Sigma HPA027524), anti-ESRP1 (210-301-B89S), anti-ESRP1/2 (210-301-C31S), anti-N-cadherin (D4R1H), anti-Met (25H2) (CST 3127) and anti-Met phospho 1234/1235 (D26) (CST 3077) were validated by western blot using specific shRNAs or inhibitors. Additional validation information is available from manufacturers.<br><br>This description of validation is included in manuscript.

Eukaryotic cell lines

Policy information about cell lines

Cell line source(s)	Parental PC3 (ATCC) and PC3 variants: PC3 E-cad+, TEM4-18, TEM2-5, GS689.Li, GS694.LAd, GS683.LALN, JD1203.Lu, GS672.Ug (M. Henry, University of Iowa), PC3-Epi and PC3-EMT (K. Pienta, Johns Hopkins School of Medicine), PC3M, PC3M-DR and also CWR (H. Leung, Beatson Institute). HEK 293-FT (Thermo Fisher Scientific), RWPE-1 and RWPE-2 cell lines (ATCC). MDCK-II (K. Mostov, UCSF) and Caco-2 cells (L. Machesky, Beatson Institute). MDA-MB-231 (M. Olson, Beatson Institute). Pdx1-Cre, LSL-KrasG12D/+ (KC), Pdx1-Cre, LSL-KrasG12D/+ LSL-Trp53R172H/+ (KPC), Pdx1-Cre, LSL-KrasG12D/+ LSL-Trp53fl/+ (KPflC) and Pdx1-Cre, LSL-KrasG12D/+, Ptenfl/+ cells (Pten) (J. Morton, Beatson Institute). Mouse derived villinCreER; KrasG12D/+; Trp53fl/fl; Rosa26N1icd/+ organoid line RBVKPN RKAC13.1g (O. Sansom, Beatson Institute).
Authentication	PC3, HEK293-FT, RWPE-1 and RWPE-2 cells were authenticated using short tandem repeat (STR) profiling in house (Promega Geneprint 10). Other cell lines were not validated by us.
Mycoplasma contamination	All cells were screened for mycoplasma contamination routinely and were negative for mycoplasma.
Commonly misidentified lines (See ICLAC register)	No misidentified cells were used in this study.
