# Supplementary material for: Traject3d allows label-free identification of distinct co-occurring phenotypes within 3D culture by live imaging
Source: Nat Commun. 2022 Sep 9;13:5317. doi: 10.1038/s41467-022-32958-x (PMC9463449; doi:10.1038/s41467-022-32958-x)
Supplement: Supplementary file 3 — Description of Additional Supplementary Files [file 41467_2022_32958_MOESM3_ESM.pdf]

Title: Supplementary Movie 1
Description: Trajectory 1. Animations of data from Supplementary Figures 9 and 10. Trajectory Motif, State Transitions, State Classification overlaid onto representative spheroids, and Area and Movement features over time.

Title: Supplementary Movie 2
Description: Trajectory 2. Animations of data from Supplementary Figures 9 and 10. Trajectory Motif, State Transitions, State Classification overlaid onto representative spheroids, and Area and Movement features over time.

Title: Supplementary Movie 3
Description: Trajectory 3. Animations of data from Supplementary Figures 9 and 10. Trajectory Motif, State Transitions, State Classification overlaid onto representative spheroids, and Area and Movement features over time.

Title: Supplementary Movie 4
Description: Trajectory 4. Animations of data from Supplementary Figures 9 and 10. Trajectory Motif, State Transitions, State Classification overlaid onto representative spheroids, and Area and Movement features over time.

Title: Supplementary Movie 5
Description: Trajectory 5. Animations of data from Supplementary Figures 9 and 10. Trajectory Motif, State Transitions, State Classification overlaid onto representative spheroids, and Area and Movement features over time.

Title: Supplementary Movie 6
Description: Trajectory 6. Animations of data from Supplementary Figures 9 and 10. Trajectory Motif, State Transitions, State Classification overlaid onto representative spheroids, and Area and Movement features over time.

Title: Supplementary Movie 7
Description: Trajectory 7. Animations of data from Supplementary Figures 9 and 10. Trajectory Motif, State Transitions, State Classification overlaid onto representative spheroids, and Area and Movement features over time.

Title: Supplementary Movie 8
Description: Trajectory 8. Animations of data from Supplementary Figures 9 and 10. Trajectory Motif, State Transitions, State Classification overlaid onto representative spheroids, and Area and Movement features over time.

Title: Supplementary Movie 9
Description: Trajectory 9. Animations of data from Supplementary Figures 9 and 10. Trajectory Motif, State Transitions, State Classification overlaid onto representative spheroids, and Area and Movement features over time.

Title: Supplementary Movie 10
Description: Trajectory 10. Animations of data from Supplementary Figures 9 and 10. Trajectory Motif, State Transitions, State Classification overlaid onto representative spheroids, and Area and Movement features over time.

Title: Supplementary Movie 11
Description: Trajectory 11. Animations of data from Supplementary Figures 9 and 10. Trajectory Motif, State Transitions, State Classification overlaid onto representative spheroids, and Area and Movement features over time.

Title: Supplementary Movie 12

Description: Trajectory 12. Animations of data from Supplementary Figures 9 and 10. Trajectory Motif, State Transitions, State Classification overlaid onto representative spheroids, and Area and Movement features over time.

Title: Supplementary Movie 13

Description: Trajectory 13. Animations of data from Supplementary Figures 9 and 10. Trajectory Motif, State Transitions, State Classification overlaid onto representative spheroids, and Area and Movement features over time.

Title: Supplementary Movie 14

Description: Trajectory 14. Animations of data from Supplementary Figures 9 and 10. Trajectory Motif, State Transitions, State Classification overlaid onto representative spheroids, and Area and Movement features over time.

Title: Supplementary Movie 15

Description: Trajectory 15. Animations of data from Supplementary Figures 9 and 10. Trajectory Motif, State Transitions, State Classification overlaid onto representative spheroids, and Area and Movement features over time.

Title: Supplementary Movie 16

Description: Trajectory 16. Animations of data from Supplementary Figures 9 and 10. Trajectory Motif, State Transitions, State Classification overlaid onto representative spheroids, and Area and Movement features over time.

Title: Supplementary Movie 17

Description: Trajectory 17. Animations of data from Supplementary Figures 9 and 10. Trajectory Motif, State Transitions, State Classification overlaid onto representative spheroids, and Area and Movement features over time.

Title: Supplementary Movie 18

Description: Trajectory 18. Animations of data from Supplementary Figures 9 and 10. Trajectory Motif, State Transitions, State Classification overlaid onto representative spheroids, and Area and Movement features over time.

Title: Supplementary Movie 19

Description: Trajectory 19. Animations of data from Supplementary Figures 9 and 10. Trajectory Motif, State Transitions, State Classification overlaid onto representative spheroids, and Area and Movement features over time.

Title: Supplementary Movie 20

Description: Trajectory 20. Animations of data from Supplementary Figures 9 and 10. Trajectory Motif, State Transitions, State Classification overlaid onto representative spheroids, and Area and Movement features over time.

Title: Supplementary Movie 21

Description: Trajectory 21. Animations of data from Supplementary Figures 9 and 10. Trajectory Motif, State Transitions, State Classification overlaid onto representative spheroids, and Area and Movement features over time.

Title: Supplementary Movie 22

Description: Trajectory 22. Animations of data from Supplementary Figures 9 and 10. Trajectory Motif, State Transitions, State Classification overlaid onto representative spheroids, and Area and Movement features over time.

Title: Supplementary Movie 23

Description: Trajectory 23. Animations of data from Supplementary Figures 9 and 10. Trajectory Motif, State Transitions, State Classification overlaid onto representative spheroids, and Area and Movement features over time.
